# The role of organic acids on microbial deterioration in the *Radix pseudostellariae* rhizosphere under continuous monoculture regimes

**DOI:** 10.1038/s41598-017-03793-8

**Published:** 2017-06-14

**Authors:** Hongmiao Wu, Linkun Wu, Quan Zhu, Juanying Wang, Xianjin Qin, Jiahui Xu, Lufei Kong, Jun Chen, Sheng Lin, Muhammad Umar Khan, Hira Amjad, Wenxiong Lin

**Affiliations:** 1 Fujian Provincial Key Laboratory of Agroecological Processing and Safety Monitoring, College of Life Sciences, Fujian Agriculture and Forestry University, Fuzhou, 350002 P. R. China; 2 Key Laboratory of Crop Ecology and Molecular Physiology (Fujian Agriculture and Forestry University), Fujian Province University, Fuzhou, 350002 P. R. China; 3 Key Laboratory for Genetics, Breeding and Multiple Utilization of Crops, Ministry of Education/College of Crop Science, Fujian Agriculture and Forestry University, Fuzhou, 350002 P. R. China

## Abstract

A three-year field monoculture trial of *Radix pseudostellariae* and complementary laboratory studies were conducted to further elucidate the underlying mechanism responsible for significant decreases in the biomass yield and quality of *R. pseudostellariae* under continuous monoculture regimes. HPLC analysis indicated that continuous monoculture soil was rich in organic acids, which had cumulative effects over time. Further analysis suggested that the application of a mixture of organic acids significantly promoted growth of pathogenic fungi, and increased the expression of chemotaxis-related gene (*che*A) and biofilm formation of the specific pathogenic *Kosakonia sacchari*. However, opposite reactions were observed in the case of *Bacillus megaterium* and *Bacillus pumilus*. Concurrently, the present results revealed that the mixed organic acids stimulated the production of toxins, as well as H_2_O_2_ in the pathogenic fungi. Furthermore, the presence of organic acids reflecting environmental conditions under monocropping had negative effects on the expression of the biocontrol-related genes, which resulted in attenuated antagonistic activities of plant growth-promoting rhizobacteria (PGPR) to suppress mycelial growth of the pathogenic fungi. These results help to unveil the mechanisms associated with how accumulated organic acids differentially mediate deterioration of soil microbial composition and structure in monocropping system.

## Introduction

Continuous crop monoculture is a common and worldwide trend in modern agriculture systems, despite the fact crop rotation has many beneficial aspects, such as enhanced soil fertility, improved physicochemical properties and protection against pests and diseases. Nevertheless, the practice of monoculturing shows an increasing trend in popularity, mainly due to government incentives, technological advances and market needs. It has been reported that the area devoted to cultivation of medicinal plants in China have increased from 0.242 million to 0.306 million ha through these production methods over the past three years. Many studies have shown that crops grown in continuous monoculture systems over time are more vulnerable to lower yields and reduced quality than those planted for the first time or in a longer rotation cropping pattern, and monoculturing eventually leads to a condition known as soil sickness or replant disease^1–4^. Previous studies have shown that more than 70% of medicinal plants have suffered from the replanting diseases, which negatively influences both soil and plant health^5, 6^. Some farmers have attempted to apply increased amounts of fertilizers and pesticides to overcome these problems and achieve higher economic returns. However, this vigorous approach to mitigating the disease fails to solve the problem, while aggravating environmental pollution and a decline in quality of the medicinal plant products from this the cropping system. Therefore, it has become a top priority to elucidate the mechanisms underlying consecutive monoculture problems, especially in the case of medicinal plant production.

**Figure 1:**
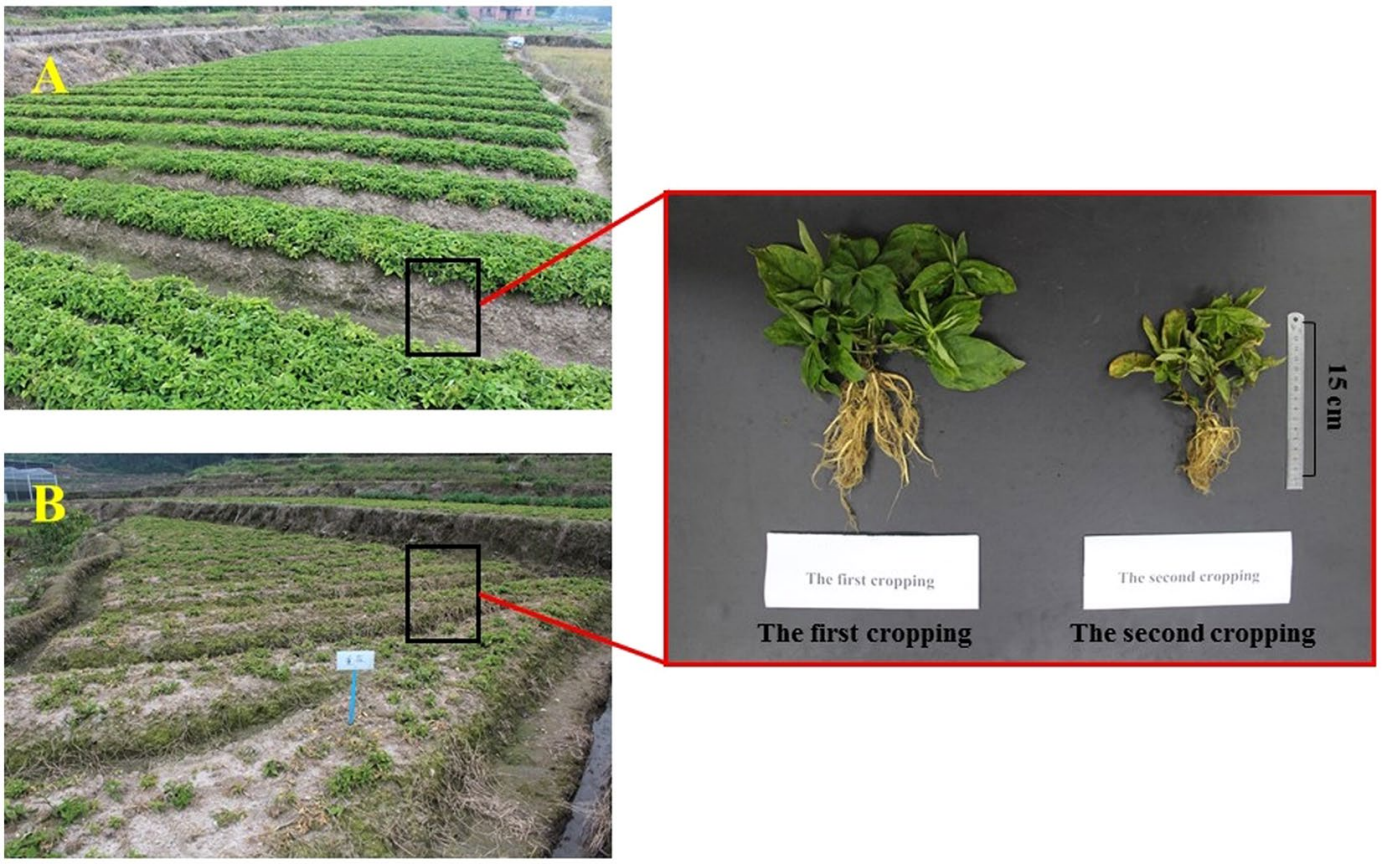
Photographs of above and below ground components of *Radix pseudostellariae* under one-year and two-year consecutive monoculture. (**A**) newly planted *R. pseudostellariae*, (**B**) two-year monocultured *R. pseudostellariae*.

*Radix pseudostellariae* L. (*Caryophyllaceaeis*) is one of the most common and highly demanded Chinese medicines in China^2^. The annual yield of this plant is typically 5000 tons, representing a value of more than 22 million dollars per year. As shown in Fig. 1, the consecutive monoculture of this crop results in a serious decline in biomass and quality of underground tubers. The results of our previous investigations have shown a significant increase in the amount of pathogenic *Fusarium oxysporum*, *Talaromyces helicus* and *Kosakonia sacchari* in the rhizosphere of *R. pseudostellariae* as the number of monoculture years increased, while the opposite trend was observed for the beneficial *Bacillus pumilus* ^2, 5, 7, 8^. In addition, a rise in the fungi/bacteria ratio was observed in soil degraded from consecutive monoculture systems^3, 4, 9^. These findings that consecutive monoculture regimes destabilize soil structure and health. However, the reasons for which consecutive monoculture causes imbalanced microbial communities are still not well understood.

Root exudates are perceived as the first line of communication between roots and microorganisms in the rhizosphere^10–12^, playing important roles in the rhizosphere dialogue between plants and microbes^13–15^. Root exudates are typically composed of amino acids, organic acids, phenolics, sugars, and proteins^10, 16^, and not only serve as carbon or nitrogen sources for soil microorganisms, but also act as signals to attract or repel microbes^17, 18^. Many studies have focused on organic acids, which represent one of the key metabolites present in root exudates of plant^19, 20^, but the role of organic acids in rhizosphere soil have not yet been clearly identified. To further understand the detailed mechanisms related to the high fungi to bacteria ratio in the rhizosphere soil of the monocropped *R. pseudostellariae*, we investigated the relationship between organic acids in the soil and microbial community structure. Subsequently, we analyzed the influence of organic acids on the physiological characteristics of pathogens (*Fusarium oxysporum*, *Talaromyces helicus*, *Kosakonia sacchari* and *Fusarium moniliforme*) and beneficial bacteria (*B. pumilus* and *Bacillus megaterium*), isolated from monocultured *R. pseudostellariae* rhizosphere soil and found to be involved in the consecutive monoculture problems. We also validated their pathogenicity and growth-promoting effects on the *R. pseudostellariae*. We determined the impact of organic acids on the production of H_2_O_2_ and toxins by these pathogenic fungi. We also evaluated chemotaxis and biofilm formation of the pathogenic and beneficial bacteria exposed to different concentrations of the organic acids being investigated. The results of this study provide useful information and insights into the molecular ecological mechanism of destabilization of soil microbial populations mediated by rhizospheric organic acids accumulated in soil under monocropping systems.

## Results

### Organic acid identification in the *R. pseudostellariae* rhizosphere and tissue culture medium

**Figure 2:**
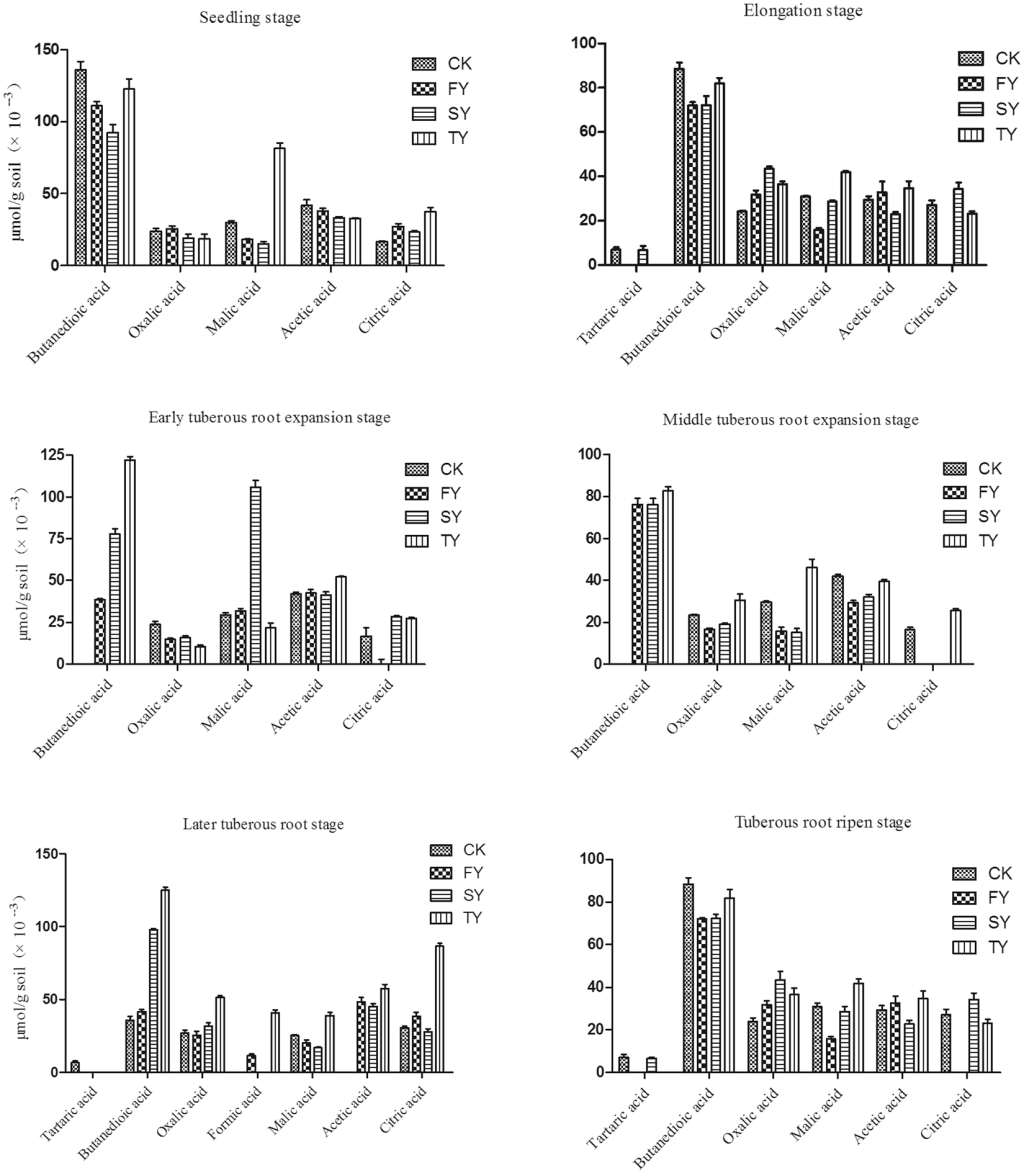
Changes in the content of organic acids in the rhizosphere soil of *R. pseudostellariae* sampled at different growth stages in a continuous cropping system. CK represents the control with no *R. pseudostellariae* cultivation. FY, SY, and TY represent the newly planted, two-year and three-year monocultured *R. pseudostellariae*, respectively, grown in fields.

The compositions of organic acids were determined in the rhizosphere soil of *R. pseudostellariae* at different growth stages during different years of monoculture. Seven types of organic acids were successfully identified in the soil. The identified organic acids were tartaric acid, butanedioic acid, oxalic acid, formic acid, malic acid, acetic acid and citric acid (Fig. 2). We also found these organic acids in the tissue culture medium of *R*. *pseudostellariae* (Figure S1). The concentrations of those organic acids in the monocultured rhizosphere soil showed a fluctuating pattern with initial increases and subsequent decreases to a certain extent in different growth stages (Fig. 2). These organic acids generally tended to accumulate in the soil and tissue culture medium of *R. pseudostellariae*, and this accumulation began during the early tuberous root expansion stage (Figure S2).

### The population size of bacteria and fungi in the rhizosphere soil of *R. pseudostellariae* under different years of monocropping regimes

**Figure 3:**
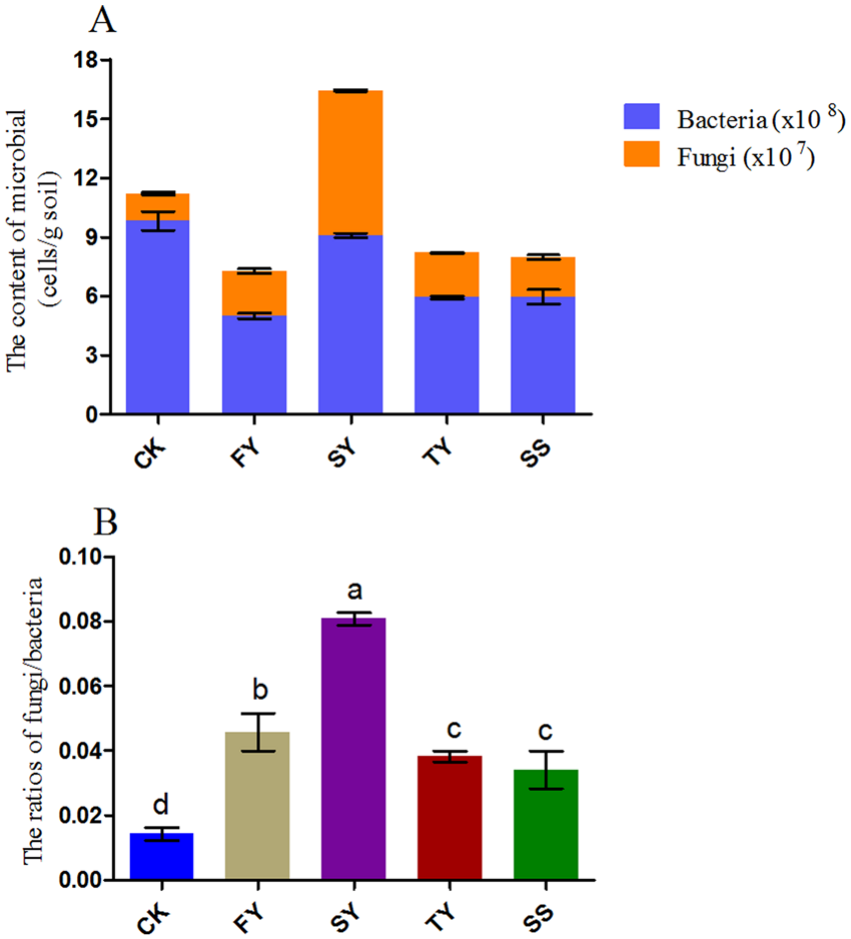
The total bacterial and fungal populations in *R. pseudostellariae* rhizosphere soil under different years of monoculture. A represents the contents of bacteria and fungi; B represents the ratios of fungi to bacteria. CK represents the control with no *R. pseudostellariae* cultivation. FY, SY, and TY represent the newly planted, two-year and three-year monocultured *R. pseudostellariae*, respectively, grown in fields. SS represents the soil around the pathogenic site of *R. pseudostellariae*. These soils were sampled from the rhizosphere soil of the fields at the same time during the later tuberous root stage of *R. pseudostellariae*. Columns with different letters are statistically different (LSD test, p < 0.05).

qRT-PCR analysis of the rhizosphere soil of *R. pseudostellariae* showed that the populations of bacteria and fungi varied with different monoculture years, with a concurrent increase in microorganism populations in the second cropping year and decrease in the third cropping year occurring for *R. pseudostellariae* monocultures (Fig. 3A). Consequently, the ratio of fungi to bacteria was significantly increased in this system (Fig. 3B). However, there was some decrease in this ratio in the third cropping year and the pathogenic site of infected *R. pseudostellariae* (SS), and these observed decreases were inconsistent with the concept that soil-borne diseases become more severe with an increase in the number of years in monoculture.

### Validation the pathogenicity and growth-promoting effects of microorganisms

The microorganisms were added to the soils with *R. pseudostellariae* grown. We validated their pathogenicity and growth-promoting effects toward *R. pseudostellariae*. The *K. sacchari*, *T. helicus*, *F. oxysporum* and *F. moniliforme* were highly pathogenic toward *R. pseudostellariae* under a one-year consecutive monoculture (Figure S3). However, *B. pumilus*, *B. megaterium* promoted the growth of *R. pseudostellariae* under two years of consecutive monoculture (Figure S3). This implies that *B. pumilus* and *B. megaterium* were beneficial bacteria in the consecutive monoculture system of *R. pseudostellariae*.

### The influence of organic acids on the physiological characteristics of pathogenic fungi

We tested the influence of several rhizospheric organic acids on the physiological characteristics of *T. helicus*, *F. oxysporum* and *F. moniliforme*. The results showed that the mixture of organic acids significantly promoted the mycelial growth of *T. helicus* and *F. moniliforme* when applied at a concentration of 120 μmol/L (Figures S4 and S5), but no positive effect on *F. oxysporum* was observed at low concentration. Further analysis revealed that various individual organic acids had different influences on mycelial growth. For example, butanedioic acid, malic acid, acetic acid significantly promoted the growth of *T. helicus* at 120 μmol/L. Oxalic acid and citric acid had no significant effects on pathogenic fungi at low doses, but exhibited an inhibitory effect at high concentrations (Figure S4).

**Figure 4:**
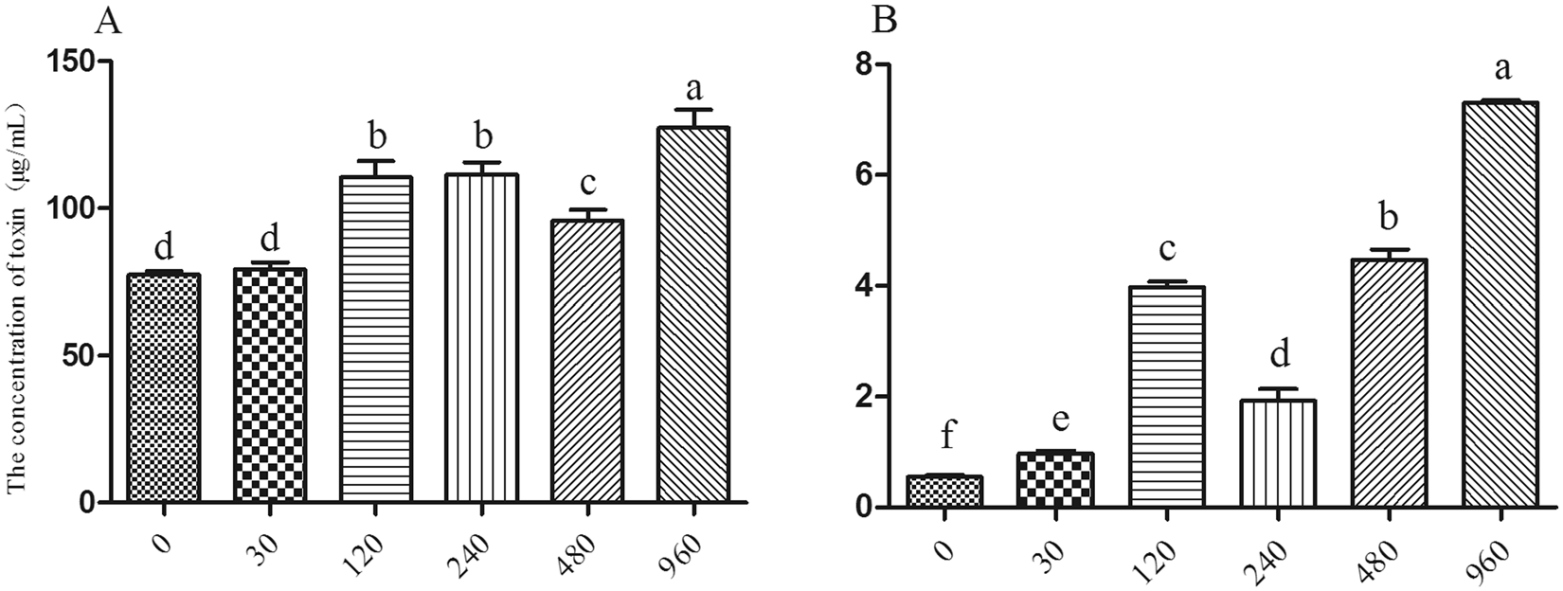
The effects of organic acid mixtures on toxin production by *T*. helicus. A represents 3A-DON; B represents 15A-DON. Columns with different letters are statistically different (LSD test, p < 0.05).

Subsequent analysis indicated that *T. helicus* could also produce two types of toxins, i.e., 3A-DON (3-Acetyldeoxynivalenol) and 15A-DON (15-O-Acetyl-4-deoxynivalenol), which inhibit the mitosis of plant cells and the mitotic index. Moreover, the concentration of the 3A-DON toxin was found to be significantly higher than that of the 15A-DON toxin (Fig. 4). Furthermore, production of the 3A-DON toxin increased in *T. helicus* cultures exposed to the organic acid mixture, and increases in production of the 15A-DON toxin were found to be directly related to the applied concentrations of the organic acid mixture, with the toxin content increasing sharply in this pathogenic fungi when exposed to a concentration of 120 μmol/L. Taken together, these findings suggested that the organic acid mixture significantly promoted the mycelial growth and production of toxins by *T. helicus* (Fig. 4).

**Figure 5:**
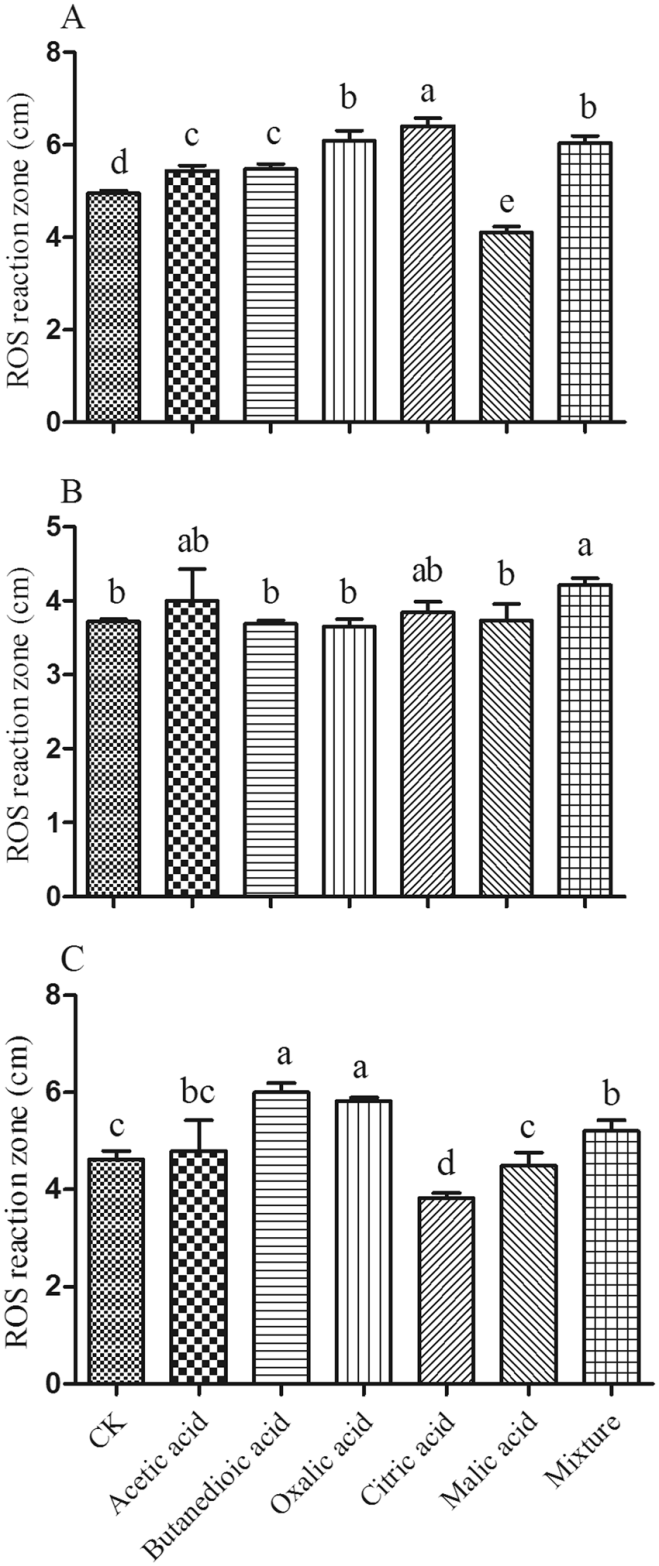
The effects of different organic acids at 120 μmol/L on hydrogen peroxide production by the fungi. (**A**) represents *F. oxysporum*; (**B**) represents *T. helicus*; (**C**) represents *F. moniliforme*. Columns with different letters are statistically different (LSD test, p < 0.05).

The mixture of the tested organic acids was also found to increase H_2_O_2_ secretion from the harmful fungi at a concentration of 120 μmol/L when compared to the control (Fig. 5). Acetic acid, butanedioic acid, oxalic acid and citric acid promoted the production of H_2_O_2_ by the pathogenic *F. oxysporum*, while butanedioic acid and oxalic acid exerted a positive effect on the production of H_2_O_2_ by the pathogenic fungus, *F. moniliforme*. However, none of the tested individual organic acids had any significant effects on *T. helicus* (Fig. 5). These findings strongly suggest that accumulation of the investigated organic acids in soil is conducive to pathogenic growth, toxin production and H_2_O_2_ secretion, which leads to an increased ability to infect host in the monoculture system.

### The effect of organic acids on the growth of bacteria

The results showed that different concentrations of the mixed organic acids had different modes of action on the pathogenic *K. sacchari*, indicating that low and high concentrations of the mixed organic acids did not have obvious effects on the specific pathogen (Figure S6). A positive effect on the growth of *K. sacchari* was observed in response to mixed acid concentrations ranging from 120 μmol/L to 480 μmol/L. Concurrently, it was found that each individual organic acids had different effects on the growth of *K. sacchari*, with tartaric acid, oxalic acid, formic acid and malic acid significantly promoting the growth of the *K. sacchari* at 120 μmol/L. Similarly, tartaric acid, oxalic acid, and formic acid also exhibited a positive effect at low concentrations, while butanedioic acid, citric acid and acetic acid had no significant effect at low concentrations, but suppressed the growth of the pathogenic bacterium *K. sacchari* at high concentrations (Figure S6). However, the mixed organic acids significantly inhibited the growth of beneficial bacteria (Figure S7), and the inhibitory effects were observed for *B. pumilus* from 30 μmol/L to 960 μmol/L, while inhibitory concentrations for *B. megaterium* ranged from 120 μmol/L to 960 μmol/L. These results further confirm that the described mixture of organic acids has a positive effect on the growth of pathogenic microorganisms, especially in pathogenic fungi, while the opposite is true in the case of beneficial bacteria, such as *B. pumilus* and *B. megaterium*.

### The chemotactic response of bacteria to organic acids

**Figure 6:**
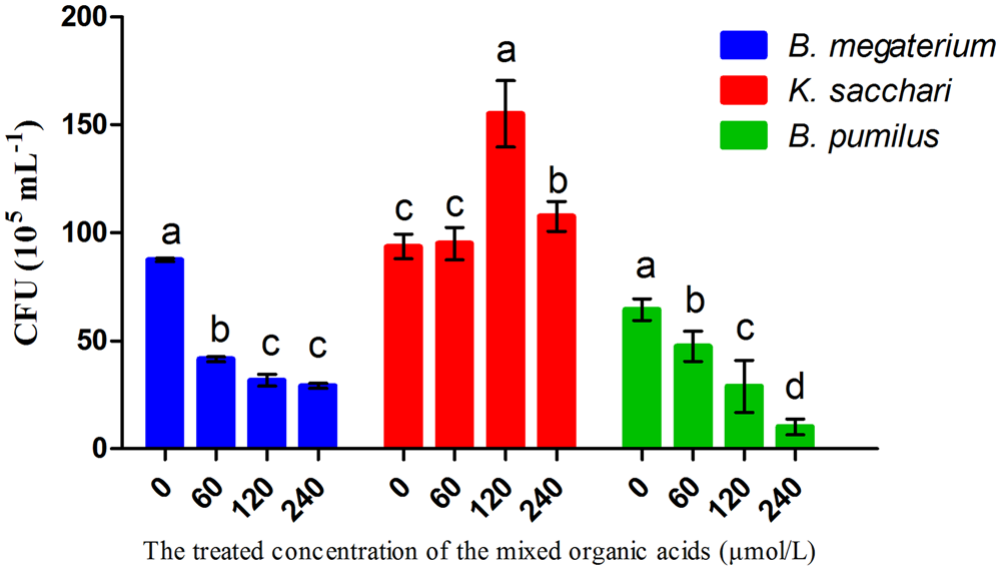
Chemotactic response of the bacteria towards the mixture of detected organic acids at different concentrations evaluated by capillary assay. Columns with different letters are statistically different (LSD test, p < 0.05).

**Figure 7:**
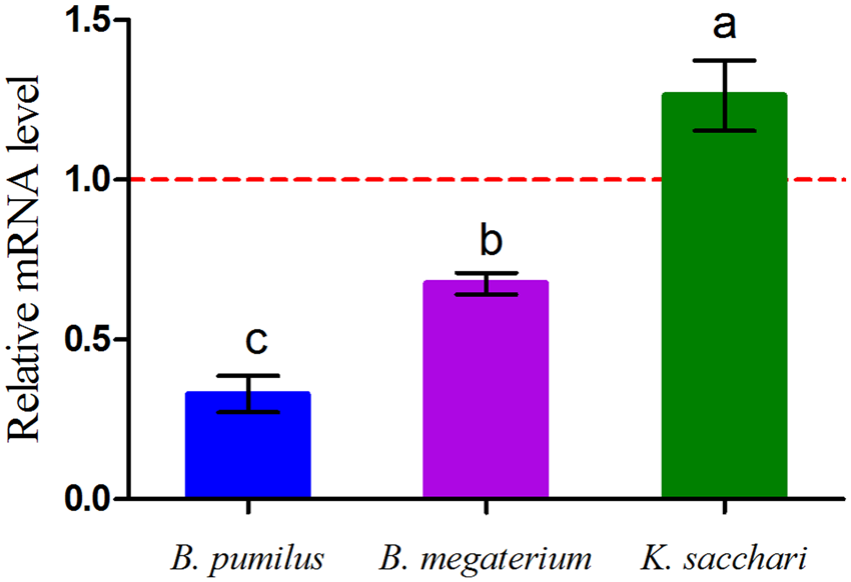
The effects of the mixed organic acids on the *che*A gene transcription in the studied bacteria. Columns with different letters are statistically different (LSD test, p < 0.05).

A capillary assay was set up to quantitatively measure the chemotactic responses of the bacteria strains to different concentrations of the mixed organic acids. Both pathogenic and beneficial microorganisms (i.e., *K. sacchari*, *B. megaterium* and *B. pumilus*) exhibited chemotaxis toward the tested organic acids in Petri dish assays (Figure S8). The mixed organic acids showed the most significant attractive effect on the pathogenic bacterium *K. sacchari* from 120 μmol/L to 240 μmol/L (Fig. 6). However, they had no significant effect on *K. sacchari* at lower treatment dosages. Conversely, a negative effect on attraction was observed for *B. megaterium* and *B. pumilus* at mixed acid concentrations ranging from 60 μmol/L to 240 μmol/L. The results also indicated that *K. sacchari* showed a higher level of gene *che*A transcription than other bacteria that received the same dosage of organic acids. The mixed organic acids inhibited the *che*A gene expression in *B. megaterium* and *B. pumilus* and promoted upregulated expression of the *che*A gene in *K. sacchari* (Fig. 7). The findings suggest that the mixed organic acids were not only conducive to the growth of pathogenic fungi, but also had the greatest positive effects on the specific pathogenic bacteria, which further explains how continuous monoculture mediates shifts in the microbial community to higher fungi to bacteria ratios in rhizosphere soils.

### The effects of the organic acids on biofilm formation by bacteria

**Figure 8:**
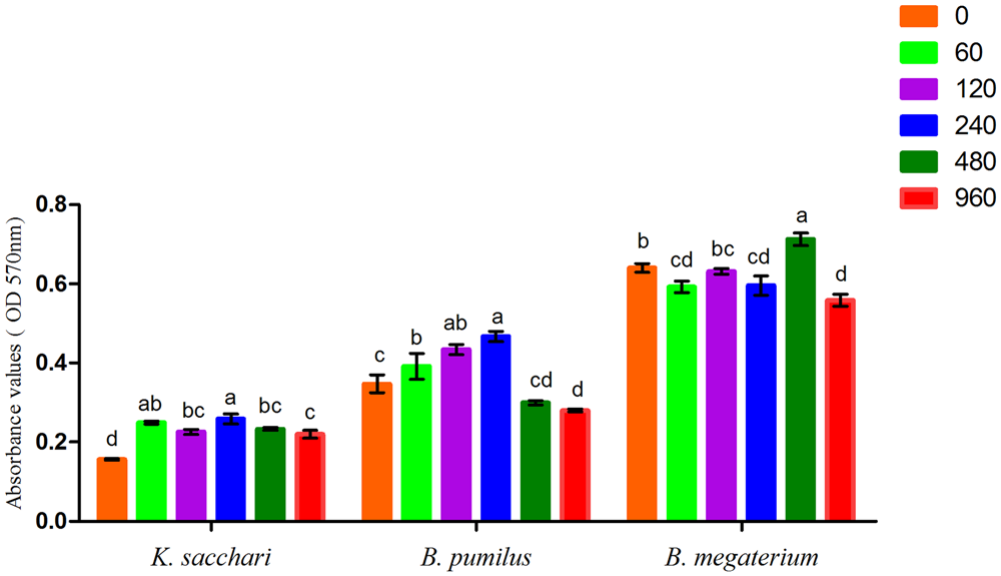
The effects of the mixed organic acids at different concentrations on biofilm formation of the bacteria. Columns with different letters are statistically different (LSD test, p < 0.05).

The results showed that the mixed organic acids significantly stimulated biofilm formation by *K. sacchari* at all dosages, while a similar effect was observed for *B. pumilus* in response to low treatment dosages. For *B. megaterium*, the mixed organic acids had a negative effect on its biofilm formation at a dosage of 240 μmol/L (Fig. 8). Further analysis revealed that various individual organic acids had different effects on biofilm formation by the tested bacteria. Specifically, butanedioic acid, oxalic acid, malic acid, acetic acid and citric acid significantly promoted biofilm formation of the pathogenic bacterium, *K. sacchari*. However, butanedioic acid and oxalic acid showed inhibitory effects on biofilm formation by *B. pumilus*, while acetic acid and malic acid had no significant effects on biofilm formation. Additionally, all individual organic acids detected in the rhizosphere soil, with the exception of acetic acid, were shown to exert a suppressive effect on biofilm formation by *B. megaterium* (Figure S9).

### The effects of organic acids on the antagonistic activity of the PGPR

**Figure 9:**
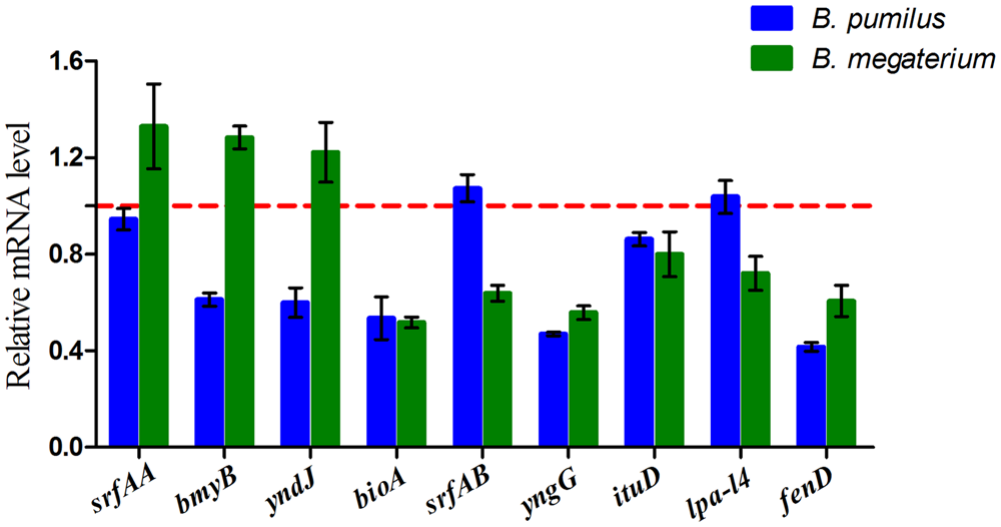
The effects of the organic acids on the transcription of biocontrol genes in bacteria evaluated by qRT-PCR.

The results showed that the PGPR (*B. pumilus* and *B. megaterium*) were able to suppress the mycelial growth of the pathogenic fungi, *T. helicus*, *F. oxysporum* and *F. moniliforme*, when they were co-cultured without the addition of any organic acids. However, the antagonistic activities were attenuated in response to increasing concentrations of the applied organic acids (Figures S10–S13). Further analysis of qRT-PCR showed that the mixed organic acids had negative effects on the transcription of many biocontrol-related genes (*srfAA*, *bmyB*, *yndJ*, *bioA*, *srfAB*, *yngG*, *ituD*, *lpa-14*, and *fenD*) in the beneficial bacteria *B. megaterium* and *B. pumilus* (Fig. 9).

## Discussion

Consecutive monoculture problem is a major problem in intensive agricultural systems in China, which alters the biogeochemistry of ecosystems and adversely affects soil biota. This problem is a typical negative plant-soil feedback condition that leads to serious declines in the biomass and quality of crops^21^. As research in this field has increased, many studies have revealed that root exudates produced by plants are able to directly or indirectly shape the rhizosphere microbiome which has some influences on the growth of plants^5, 22, 23^. Subsequent detection of organic acids in the rhizosphere of monocropped *R. pseudostellariae* revealed that some of the low molecular weight organic acids accumulated in the soil as monocropping years increased. Moreover, the MIC (minimum inhibitory concentration) value of the organic acids for pathogenic bacteria (*K. sacchari*) was approximately 8400 μmol/L, which was more than two times higher than that for the beneficial bacteria (*B. pumilus*, *B. megaterium*) (Table S1). However, the MIC values for the pathogenic fungi (*F. oxysporum*, *F. moniliforme* and *T. helicus*) were more than 36,000 μmol/L (Table S1), which was consistent with the results of previous studies^24^. These findings indicate that the accumulation of organic acids have positive effects on the proliferation of pathogenic fungi.

Chemotaxis and colonization are the two primary elements of plant-microbe interactions, with chemotaxis of bacteria towards root exudates suggested as the first step of bacterial colonization^15, 25^. Furthermore, effective colonization can be confirmed visually by the formation of a bacterial biofilm on plant roots^26, 27^. Some studies have revealed that organic acids significantly induced chemotaxis and biofilm formation in the PGPR at low concentrations (50 μmol/L)^12, 15^. Based on our results, organic acids significantly promoted the chemotactic response of the pathogenic bacterium *K. sacchari*, but inhibited the chemotactic response of the beneficial bacteria *B. pumilus* and *B. megaterium* when they were exposed to acid concentrations of 120 μmol/L, which is close to the concentration of organic acids detected in the rhizosphere soil of *R. pseudostellariae*. Moreover, while the organic acids had a positive influence on biofilm formation of the pathogenic bacterium *K. sacchari*, the opposite was true in the case of the beneficial bacteria, such as *Bacillus sp*., which has demonstrated promising effects in biocontrol for plant disease^28–30^. These results suggest that the organic acids accumulated in the rhizosphere soil are not conducive to the colonization of PGPR and significantly reduce its antagonistic ability against specific pathogens in the monoculture system. The findings further explain how root exudates are able to differentially mediate the shifts of microbial soil communities, consequently leading to increases in fungi, as well as reductions in bacteria, which in turn results in a higher fungi/bacteria ratio, thereby shifting the composition and structure of microbial flora in long-term monocropping systems.

Our results also showed that the tested mixture of concerned organic acids significantly increased the H_2_O_2_ secretion from the tested pathogenic fungi *F. oxysporum, F. moniliforme* and *T. helicus*, while concurrently promoting the production of 3A-DON and 15A-DON toxins by *T. helicus*. It is worth mentioning that these increased toxins from *T. helicus* could trigger a cascade reaction promoting the growth of the other pathogenic bacteria such as *K. sacchari*, while inhibiting the growth of beneficial bacteria such *B. pumilus*.^5^ This occurs when they were exposed to those toxins in the consecutively monocropping soil mainly due to the accumulation of organic acids in the rhizosphere soil.

This rhizospheric biological process, known as allelopathy, may be associated with reduced growth in monoculture stands over time. These allelopathic inhibitions between plants have broad implications for change in rhizosphere community structures, potentially influencing microbial coevolution and consecutively monocropping agricultural systems including weed control^31^. Allelopathy can operate directly or indirectly between the donor and recipient plant or between crop residue and the evolution of associated microflora in the rhizosphere^32, 33^. Liu *et al*.^34^ elucidated the direct allelopathy mechanism in which benzoic acid significantly increased the abundance of bacteria and fungi, but reduced the ratio of bacteria to fungi in the soil of peanut crops. The present study has focused on the autoallelopathy mediated by the root exudates and secondary metabolites of the microbes, and microbial feeding on these allelochemicals under continuous monoculture regimes. Defensive mechanisms against pathogens in many animals and plants involve the direct action of reactive oxygen species (ROS), such as hydroxyl radical (OH^•^), superoxide (O_2_^−^) and hydrogen peroxide (H_2_O_2_)^35–37^. However, pathogens can trigger increases in reactive oxygen species (ROS) known as ‘oxidative bursts’, which result in the accumulation of ROS in tissues of the plant proximal to the pathogen^37, 38^, leading to damaged cells or cell death by peroxidizing lipids and disrupting structural proteins, enzymes and nucleic acids in the host plant root tissues^38^. The results in the present study increase our understanding of autoallelopathy associated with replant problem.

**Figure 10:**
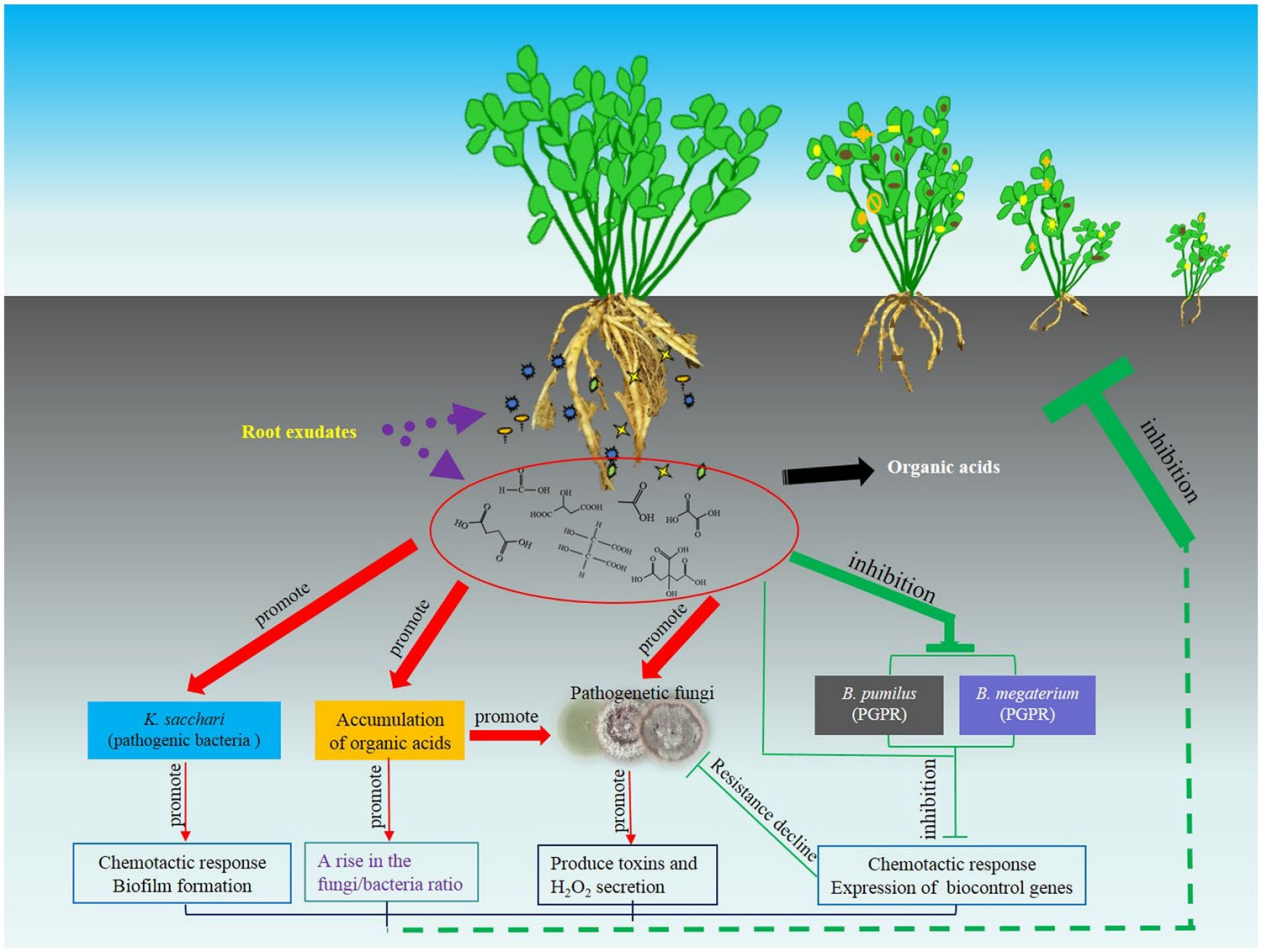
Tentative graphic of the communication between root exudates and microorganisms.

Our previous studies have shown that the phenolic exudates mediate microflora shifts and structure disorder in the rhizosphere soil of continuously monocultured *R. pseudostellariae* and *Rehmannia glutinosa*, which leads to increased incidence of disease^1, 2^. However, root exudates contain many secondary metabolites, which have different effects on plants and microorganisms. Most of these plant’s own metabolites act as autoallelopathic agents to mediate the local rhizosphere organisms. More than 30 years ago, Hartung *et al*.^39–41^ reported that allelochemicals of asparagus may have direct physiological and biochemical effects on asparagus plants that predisposes them to *Fusarium* diseases. In recent years, allelochemicals have been isolated and identified in various plants by many researchers, and these compounds have been found to include organic acids, phenolic acids, plant volatiles, terpenoids, coumarins, flavonoids, and strigolactones^1–3, 12, 15, 34, 42–44^. However, most studies conducted to date have focused on the effects of single allelochemical on the microbial community structure and/or one particular microbial kingdom. In the present study, the relationship among the plant, PGPR and pathogen have been clarified by modification of the organic acids. We provide here a case study showing a possible molecular ecological mechanism (Fig. 10) that may further explain the reason for the imbalance between pathogenic microorganisms and PGPR strains in the rhizosphere soil of *R. pseudostellariae*. These effects could be attributed to the induction of chemotaxis, biofilm formation, toxins production and H_2_O_2_ secretions in the pathogenic microorganisms mediated by the organic acids accumulated in soil under monoculture systems. This negative feedback reaction exhibited in the allelopathic interaction is involved in a vicious cycle that leads to autoallelopathy via the role organic acid secretion plays on toxins and H_2_O_2_ production, chemotaxis response and biofilm formation. The downregulated expression of biocontrol-related genes in turn leads to increased populations of pathogenic fungi, as well as destabilized composition and structure of microbial flora with higher fungi/bacteria ratios, which consequently results in significant growth inhibition of *R*. *pseudostellariae* in continuous monoculture systems.

## Conclusions

The root exudates of *R. pseudostellariae* can adversely impact the microbial community in the rhizosphere, including hosting specific pathogens at the expense of beneficial microorganisms. We found the effects of accumulated organic acids was invoked as a driver of the changes seen in the rhizosphere soils. Organic acids suppressed the growth, effective colonization and antagonistic activities of PGPR under the continuous monoculture. However, the proliferation of pathogens (*F. oxysporum, F. moniliforme*, *T. helicus* and *K. sacchari*) was mediated by organic acids. We found that the replanting diseases were caused by autoallelopathy mediated by the root exudates, which caused decreased biomass yield in *R. pseudostellariae*. Our results will open a new avenue for modulating the root microbiome to enhance crop production and sustainability.

## Materials and Methods

### Field experiment

*R. pseudostellariae* cultivar ‘Zherong-2’, which has been planted in the main production region, was used as the test plant material. The field experiment was conducted at Fuding, Fujian Province (27° 25′N, 120° 03′E). Soil samples were collected from the rhizosphere of the medicinal plants at different growth stages (seedling stage, elongation stage, early tuberous root expansion stage, middle tuberous root expansion stage, later tuberous root stage, tuberous root ripen stage) in each of the different treatments, which included the newly planted plants (FY), the two-year (SY) and three-year (TY) monocultured *R. pseudostellariae* and control treatment without planting (CK). Fresh soil samples were collected on February 22, March 22, April 22, May 5, June 6 and June 26 in 2014. The sampled soil was sieved (2 mm mesh) to remove stones and plant residues, after which the soil samples were stored at −80 °C.

### Extraction of organic acids from soil and tissue culture medium of *R. pseudostellariae*

Soil organic acids of each sample were extracted using modified methods^45, 46^. Briefly, 10 g of each soil sample were added to 15 mL double distilled water and agitated for 24 hours on a reciprocal shaker at 30 °C, then spun in a vortex generator for 30 minutes at maximum speed. The suspension was then subjected to ultrasound at 55–60 °C for 90 minutes, after which the suspension was centrifuged at 10,000 rpm for 10 minutes and the liquid supernatant was collected. Finally, the supernatant was dissolved in 15 mL of double distilled water and maintained in dark at 4 °C.

The tissue cultures of *R. pseudostellariae* were incubated for 0, 90, and 150 d. Each treatment contained 30 mL of medium. Then, the used medium was collected for extraction of the organic acids. Each sample was added to 25 mL of double distilled water and agitated for 90 min in an ultrasonic generator. The resultant extracts were pooled and evaporated to dryness by the Centrifugal Concentrators (Labconco CentriVap, USA). Each treatment was replicated 3 times. Then the supernatant was dissolved in 4 mL using double distilled water and maintained in dark at 4 °C.

### Determination of organic acids

The water solution of the extracts was filtered through 0.22 μm filter membranes for HPLC analysis. The contents of organic acids in each soil sample were determined using a Waters HPLC system (C18 column: Inertsil ODS-SP, 4.6 × 250 mm, 5 μm). Mobile phase A was methanol and mobile phase B was 5‰ potassium dihydrogen phosphate (pH 2.5). The flow rate was kept constant at 0.6 mL/min, with ultraviolet detection performed at 214 nm. The injection volume was 20 μL and the column temperature was maintained at 25 °C. Identification and quantification of organic acids were accomplished by comparing the retention times and areas with those of pure standards.

### Microorganisms

All the isolates of *Fusarium* and *Bacillus* were isolated from the rhizospheric soil (clayey soil) of monocultured *R. pseudostellariae*. The pathogenic fungi *Fusarium oxysporum* and *Fusarium moniliforme* were isolated by the selective medium^47^. The nutrient agar medium^48^ were used to isolate the plant growth promoting rhizobacteria (PGPR) *Bacillus megaterium* (KU517717) and *Bacillus pumilus* (KU517715). Concurrently, the pathogenic fungus *Talaromyces helicus* (KU355274) and the pathogenic bacterium *Kosakonia sacchari* (KU324465) were isolated from infected tubers of *R. pseudostellariae* by MS medium^5^. We found a significant increase in the amount of pathogenic *F. oxysporum*, *T. helicus* and *K. sacchari* in the rhizosphere of *R. pseudostellariae* as the number of monoculture years increased^2, 5^, while the beneficial *B. pumilus* showed the opposite trend^5^. These microorganisms have been shown to be involved in the consecutive monoculture problems of *R. pseudostellariae* ^2, 5^.

### Quantitative PCR analysis of the total bacteria and fungi in the monocultured rhizosphere soil

A quantitative PCR assay was used to determine the size of the bacterial and fungal populations in the rhizosphere soil of *R. pseudostellariae* at different cropping years. The primer sets ITS1F/ITS4^49, 50^ and Eub338/Eub518^51, 52^ were used to estimate the fungi and the bacterial community sizes, respectively. Quantitative PCR analysis was performed based on the method described by Wu *et al*.^5^ in 15 μL reaction mixtures containing 7.5 μL 2 × SYBR green I SuperReal Premix (Transgen, Beijing, China), 0.6 μL of each primer (10 μM) and template DNA (20 ng of total soil DNA or a serial dilution of plasmid DNA for standard curves). The taxon-specific primer sets and their annealing temperatures are listed in Tables S2 and S3. Four independent quantitative PCR assays were performed for each treatment.

### Effect of organic acids on colony diameter of the *R. pseudostellariae* pathogenic fungi

To prepare a soil extract-medium (SEM), 1 kg soil and 1 L double distilled water were shaken for 30 min, then sterilized at 121 °C for 15 min. Subsequently, the leachate was filtered with talc in a double suction filter. The filtrate was then collected and adjusted to neutral pH as stock solution for further use. Double distilled water was used to dilute the soil solution with appropriately diluted replicates, after which an appropriate amount of agar (15 g/L) was added to the solution, which was sterilized before use.

Based on the measured results of organic acids in the *R. pseudostellariae* rhizosphere soil, a concentration gradient series of organic acids was prepared at 0, 30, 60, 120, 240, 480, and 960 μmol/L. After sterilization, SEM medium that had been 10-fold diluted was cooled to 40–50 °C, after which a series of stock organic acid solutions dissolved in double distilled water were filtered through a 0.22 μm membrane, added to the media and immediately plated. The control received double distilled water instead of organic acids and each treatment had three replicates. Following preparation of the organic acids-SEM plates, activated *Talaromyces helicus* spores were inoculated in the center of each plate, then placed in an incubator at 30 °C constant temperature for 9 days, with the final mycelium diameter recorded being taken at the end of the experiment.

The standardized organic acids used in this study to form a mixed solution with organic acids concerned (O1) simulates the average ratio of various organic acids detected in *R. pseudostellariae* rhizosphere soil (FY, SY, TY,) (i.e., butanedioic acid: oxalic acid: malic acid: acetic acid: citric acid = 52:15:17:22:6) based on the soil measurements of the organic acids in the *R. pseudostellariae* rhizosphere. A series of concentration gradients of the mixed organic acids and single organic acids in the solution were set at 0, 30, 60, 120, 240, 480, and 960 μmol/L. The spores of *T. helicus*, *F. oxysporum* and *F. moniliforme* that had been activated were inoculated in the center of each plate. The other conditions were the same as those mentioned above.

### The impact of the mixed organic acids on toxin production by the *R. pseudostellariae* biotype *T. helicus*

The dilution ratio of the soil filtrate and its preparation were the same as those mentioned above, and the concentration gradients of the mixed organic acids were 0, 30, 120, 240, 480, and 960 μmol/L. An equal amount of *T. helicus* spores filtrate was added to the SEM liquid medium in a flask and subsequently incubated at 30 °C on a 180 rpm thermostatic shaker for 8 days. After culture, HPLC was used to determine the content of three common toxins (DON: deoxynivalenol; 3A-DON: 3-Acetyldeoxynivalenol; and 15A-DON: 15-O-Acetyl-4-deoxynivalenol). The detailed determination and extraction assay were performed based on the method described by Wu *et al*.^2, 5^.

### The impact of organic acids on the H_2_O_2_ production by the pathogenic fungi

The plates contained media consisting of the 10-times diluted SEM medium, 1.5% agar, 0.5% glucose and different kinds of organic acids. The fungal spores were inoculated in the center of the plate, then incubated at 30 °C for 10 days, with subsequent measurement of the secretion and accumulation of ROS as H_2_O_2_.

To visualize the H_2_O_2_ in the agar, the plates were stained by flooding with 6 ml of 100 mM potassium phosphate buffer, pH 6.9, 2.5 mM 3,3′-diaminobenzidine tetrachloride (DAB) and 5 purpurogallin units ml^−1^ of horseradish peroxidase (Type VI-A), swirled to cover the entire surface, and incubated at room temperature for 10 hours^53, 54^. To stop the color development, plates were rinsed in sterile ddH_2_O, after which the ROS reaction zones were measured for each plate.

### The influence of organic acids on the physiological characteristics of the specific bacteria

The LB liquid culture medium was diluted 6 times, placed into glass tubes, and subjected to a high-temperature condition of 121 °C and high pressure sterilization for 20 min. When the culture medium was sufficiently cooled, an appropriate amount of each organic acid’s stock solution (i.e., tartaric acid, butanedioic acid, oxalic acid, formic acid, malic acid, acetic acid and citric acid) was added to the tubes. Subsequently, active bacterial (*K. sacchari, B. pumilus* and *B. megaterium*) liquid culture was added to each tube (30 μL), and all tubes were incubated at 37 °C on a thermostatic shaker at 200 rpm for 8 to 10 h. Finally, 200 μL of bacterial liquid culture was transferred to a 96-well-culture-cluster, and the multi-functional plate reader SpectraMax i3 analysis system (Multi-Mode Detection Platform, USA) was used to determine the absorbance values at OD 600 nm.

Based on solution O1, the final concentration gradients of each mixture of organic acids were: 0, 30, 60, 120, 240, 480, and 960 μmol/L. Other conditions were the same as those mentioned above.

### Screening of the chemotactic bacteria via a Petri-dish and capillary assay

Bacterial chemotaxis was tested using a soft-agar swarm plate assay, with organic acid mixtures containing butanedioic acid, oxalic acid, malic acid, acetic acid and citric acid as the chemotactic attractants and 0.3% agar. Briefly, bacteria were grown in LB medium at 37 °C and 200 rpm. At the log phase (OD600 = 0.4), bacterial cells were harvested and washed three times with MM solution^55, 56^, then resuspended in the same MM solution (OD600 = 0.4). The mixed organic acids (final concentration 120 μmol/L) were added to the swarm plate medium before pouring into the plates. Next, 50 μL cell suspension containing 1 mM glucose were gently poured onto the center of the plate and incubated at 37 °C. All treatments were performed in triplicate.

A modified capillary assay was performed based on the method described by Adler *et al*.^57^ and Yuan *et al*.^12^ to quantitatively determine the chemotaxis response of the bacteria to the organic acids components. The bacteria were grown in LB media until an OD600 of 0.6 was reached, after which the cells were collected by centrifugation and washed twice with the chemotaxis buffer (100 mM potassium phosphate [pH 7.0] with 20 μM EDTA), then resuspended in the same buffer (OD600 = 0.6). A 90 mm Petri-dish was then filled with 20 mL of the cell suspension prepared above. Standard 10 μL capillaries were then loaded with the mixed organic acids at different concentrations (0, 60, 120, and 240 μmol/L) and immersed in the cell suspension in the Petri-dishes. After 30 min of incubation at RT, the liquid in the capillary was transferred into a sterilized Eppendorf tube via syringe. The suspension was subsequently diluted to 10^−3^ and 10^−4^ and plated on LB plates. Finally, the CFUs were determined by plating on LB plates and incubating at 37 °C for 24 h. Each treatment was replicated three times.

### Biofilm formation assay

To determine the effects of the organic acids on bacterial biofilm formation, an assay was performed in 96-well microtiter plates as previously described^12, 58^. The bacterial strains were grown in LB medium at 37 °C until an OD600 of 0.8 was reached, after which the cells were centrifuged, washed, and finally resuspended in 1/2 MSgg at the same volume as the culture medium. Each well was filled with 250 μL 1/2 MSgg media and inoculated with 10 μL of the suspension prepared above. The negative control consisted of the culture medium alone. The mixture of organic acids (final concentration 0, 60, 120, 240, 480, and 960 μmol/L) was added to the media in the wells. Individual organic acids (i.e., butanedioic acid, oxalic acid, malic acid, acetic acid, and citric acid) were also added to the media to obtain a final concentration of 120 μmol/L for each acid, with each treatment replicated eight times. Following incubation at 37 °C for 32 h, the biomass of the biofilms formed by the bacteria was harvested from the 96-well plate and washed with distilled water. These attached cells were then stained with 250 μL of 0.1% crystal violet for 30 min at RT, after which the excess crystal violet was poured out and the wells were washed twice with distilled water. The bound crystal violet was further solubilized with 250 μL of 4:1 (v:v) ethanol and acetone. To quantify the biofilm formation, a multi-functional plate reader SpectraMax i3 analysis system (Multi-Mode Detection Platform, U.S.A) was used to measure OD570 of the solution in each well.

### Transcription of chemotaxis and biocontrol genes under treatments with the mixed organic acids

The 5 mL culture medium contained a 6-fold dilution of the LB liquid, as well as the mixture of organic acids (final concentration 120 μmol/L). Finally, activated liquid cultures of *K. sacchari*, *B. pumilus* and *B. megaterium* liquid (50 μL) were added to each tube, after which they were incubated at 37 °C on a thermostatic shaker at 200 rpm for 8 h.

Total RNA samples were extracted using an RNAiso Plus kit (TaKaRa, Dalian, China) based on the manufacturer’s protocol. Next, 20 μL samples were analyzed on a reverse transcription system (Transgen, Beijing, China) according to the manufacturer’s protocols to reverse transcribe the isolated RNA into cDNA. Quantitative reverse transcription PCR was then applied to evaluate the transcription levels of the chemotaxis-related genes *che*A of the three kinds of bacteria as previously described^59^, and the transcription levels of the biocontrol-related genes which included the *srfAA*, *bmyB*, *yndJ*, *bioA*, *srfAB*, *yngG*, *ituD*, *lpa-14* and *fenD* ^29, 60, 61^ of beneficial bacteria strains, *B. pumilus* and *B. megaterium*. These genes acted as the lipid peptide antibiotic synthesis gene (*srfAA*, *bmyB*, *ituD*), biotin synthase gene (*bioA*), antibacterial protein synthesis gene (*yndJ*) and lipid peptide antibiotic family (*lpa-14*, *srfAB*, *yngG*) in the *Bacillus sp*. All primers and parameters involved in the experiment are listed in Tables S2 and S3.

### Validating the antagonistic activity of PGPR against the pathogenic fungi under treatment with the mixed organic acids

The amended agar disk diffusion method^62^ was used to qualitatively screen the antagonists. The culture medium consisted of 1/4 PDA and a series of concentration gradients of the mixed organic acids. Briefly, three strains of PGPR were streaked onto the medium with the same distance to the center of the plate. The pathogenic fungi were then removed from actively growing colony margins of *T. helicus*, *F. oxysporum* and *F. moniliforme* and placed in the center of the plate. The plates were then incubated for 2 d at 37 °C, followed by an additional 5 d at 30 °C.

### Statistical analysis

Differences among treatments were calculated and statistically analyzed by analysis of variance (ANOVA) and the LSD multiple range test, with a p < 0.05 taken to indicate significance. The Statistical Package for the GraphPad Prism version 5.1 and the Data Processing System (DPS) version 7.05 were used for statistical analysis.

## Electronic supplementary material

Supporting information
