# Supplementary material for: The role of organic acids on microbial deterioration in the Radix pseudostellariae rhizosphere under continuous monoculture regimes
Source: Sci Rep. 2017 Jun 14;7:3497. doi: 10.1038/s41598-017-03793-8 (PMC5471291; doi:10.1038/s41598-017-03793-8)
Supplement: Supplementary file 1 — Supporting information [file 41598_2017_3793_MOESM1_ESM.doc]

**The role of organic acids on** **microbial** **deterioration in the *Radix pseudostellariae* rhizosphere under continuous monoculture regimes**

Hongmiao Wu^1,2^, Linkun Wu^1,2^, Quan Zhu^1,2^, Juanying Wang^1,2^, Xianjin Qin^2,3^, Jiahui Xu^1,2^, Lufei Kong^1,2^, Jun Chen^1,2^, Sheng Lin^1,2^, Muhammad Umar Khan^1,2^, Hira Amjad^1,2^, Wenxiong Lin^1,2,3*^

^1^ Fujian Provincial Key Laboratory of Agroecological Processing and Safety Monitoring, College of Life Sciences, Fujian Agriculture and Forestry University, Fuzhou 350002, PR China.

^2^ Key Laboratory of Crop Ecology and Molecular Physiology (Fujian Agriculture and Forestry University), Fujian Province University, Fuzhou 350002, PR China.

^3^ Key Laboratory for Genetics, Breeding and Multiple Utilization of Crops, Ministry of Education / College of Crop Science, Fujian Agriculture and Forestry University, Fuzhou 350002, PR China

**Additional information**

**Tables**

**Table S1 The MIC (minimum inhibitory concentration) of organic acids on the different microorganism**

|  | *K. sacchari*, | *B. pumilus* | *B. megaterium* | *T. helicus* | *F. oxysporum* | *F. moniliforme* |
| --- | --- | --- | --- | --- | --- | --- |
| Mixed organic acids (μmol/L) | 8400 | 3600 | 3600 | 36000 | 36000 | 48000 |

**Table S2** Primers used for quantitative reverse transcription PCR in this study

| Gene | Name | Sequence | Reference |
| --- | --- | --- | --- |
| ITS | ITS1F | CTTGGTCATTTAGAGGAAGTAA |  |
|  | ITS4 | TCCTCCGCTTATTGATATGC |  |
| Eub | Eub338 | ACTCCTACGGGAGGCAGCAG |  |
|  | Eub518 | ATTACCGCGGCTGCTGG |  |
| *che*A | P4P5F | GGIMGIGGIGTIGGIATGGAYGTIGT |  |
|  | P4P5R | CCRTCICCIARIATIGTIGCICC |  |
| *srfAA* | SRFAF | GAAAGAGCGGCTGCTGAAAC |  |
|  | SRFAR | CCCAATATTGCCGCAATGAC |  |
| *bmyB* | BMBF | TGAAACAAAGGCATATGCTC |  |
|  | BMBR | AAAAATGCATCTGCCGTTCC |  |
| *yndJ* | yndJF | CAGAGCGACAGCAATCACAT |  |
|  | yndJR | TGAATTTCGGTCCGCTTATC |  |
| *bioA* | bioAF | TTCCACGGCCATTCCTATAC |  |
|  | bioAR | TTTGTCCCCTTATCCTGCAC |  |
| *srfAB* | srfABF | GTTCTCGCAGTCCAGCAGAAG |  |
|  | srfABR | GCCGAGCGTATCCGTACCGAG |  |
| *yngG* | yngGF | GAACTGTCCGAA ACATGTCCG |  |
|  | yngGR | CTGAGCTCTTGAACGGTCCGG |  |
| *ituD* | ituDF | ATGAACAATTTGCCTTTTTA |  |
|  | ituDR | TTATTTTAAAATCCGCAATT |  |
| *lpa-14* | lpa-14F | ATGAAAATTTACGGAGTATA |  |
|  | lpa-14R | TTATAACAGCTCTTCATACG |  |
| *fenD* | FNDF | CCTGCAGAAGGAGAAGTGAAG |  |
|  | FNDR | TGCTCATCGTCTTCCGTTTC |  |
|  | rrsw-F1 | CGGGGAGGAAGGTGTTGTG | This study |
|  | rrsw-R1 | GAGCCCGGGGATTTCACATC |  |
|  | Bp-irR | CCGCGTGAGTGATGAAGGTT | This study |
|  | Bp-irF | GGACAACGCTTGCCACCTAC |  |
|  | BMR2 | GCCGTACTCAGGATCCACTC | This study |
|  | BMF2 | AAGGTCCGTCGAAGAAGGTA |  |

**Table S3** The qRT-PCR parameters used in this study

| Gene name Reactions conditions Reference | | | | |
| --- | --- | --- | --- | --- |
| ITS1F/ITS4 | 94°C for 45s | 51°C for 45s | 72°C for 60s | This study |
| Eub338/Eub518 | 95°C for 20s | 53°C for 20s | 72°C for 20s |  |
| ituDF/ ituDR | 95°C for 10s | 48°C for 30s | 72°C for 20s |  |
| lpa-14F/ lpa-14R |  | 52°C for 30s |  |  |
| P4P5F/P4P5R |  | 55°C for 30s |  |  |
| SRFAF/SRFAR  BMBF/BMBR  yndJF/yndJR  bioAF/bioAR |  | 56°C for 30s |  |  |
| FNDF/FNDR  Bp-irR/Bp-irF  BMR2/BMF2 |  | 60°C for 30s |  |  |
| srfABF/srfABR  yngGF/yngGR |  | 63°C for 30s |  |  |


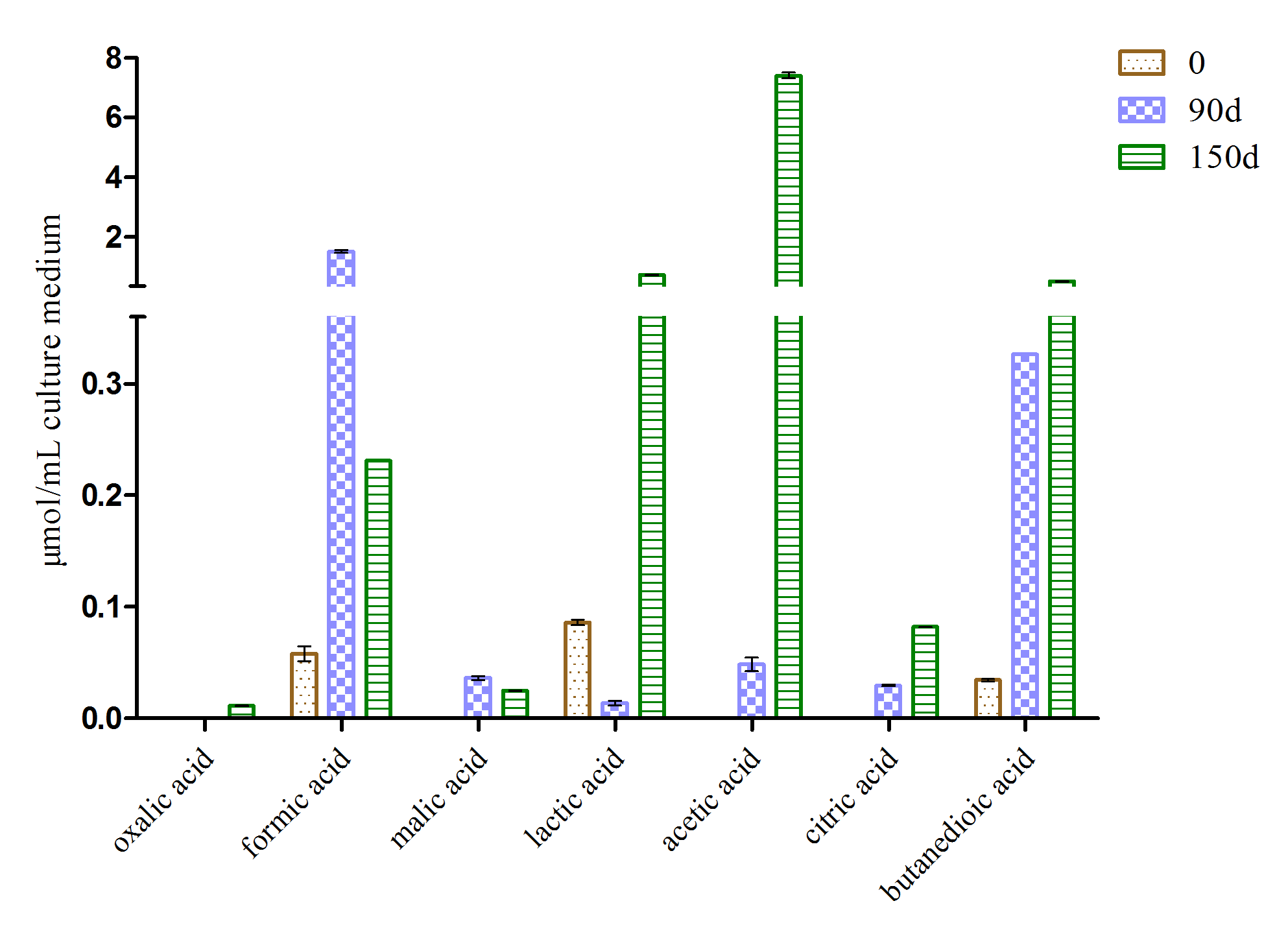
**Figure S1** variety in the contents of the organic acids in the tissue culture medium of *R. pseudostellariae* on different growth days.


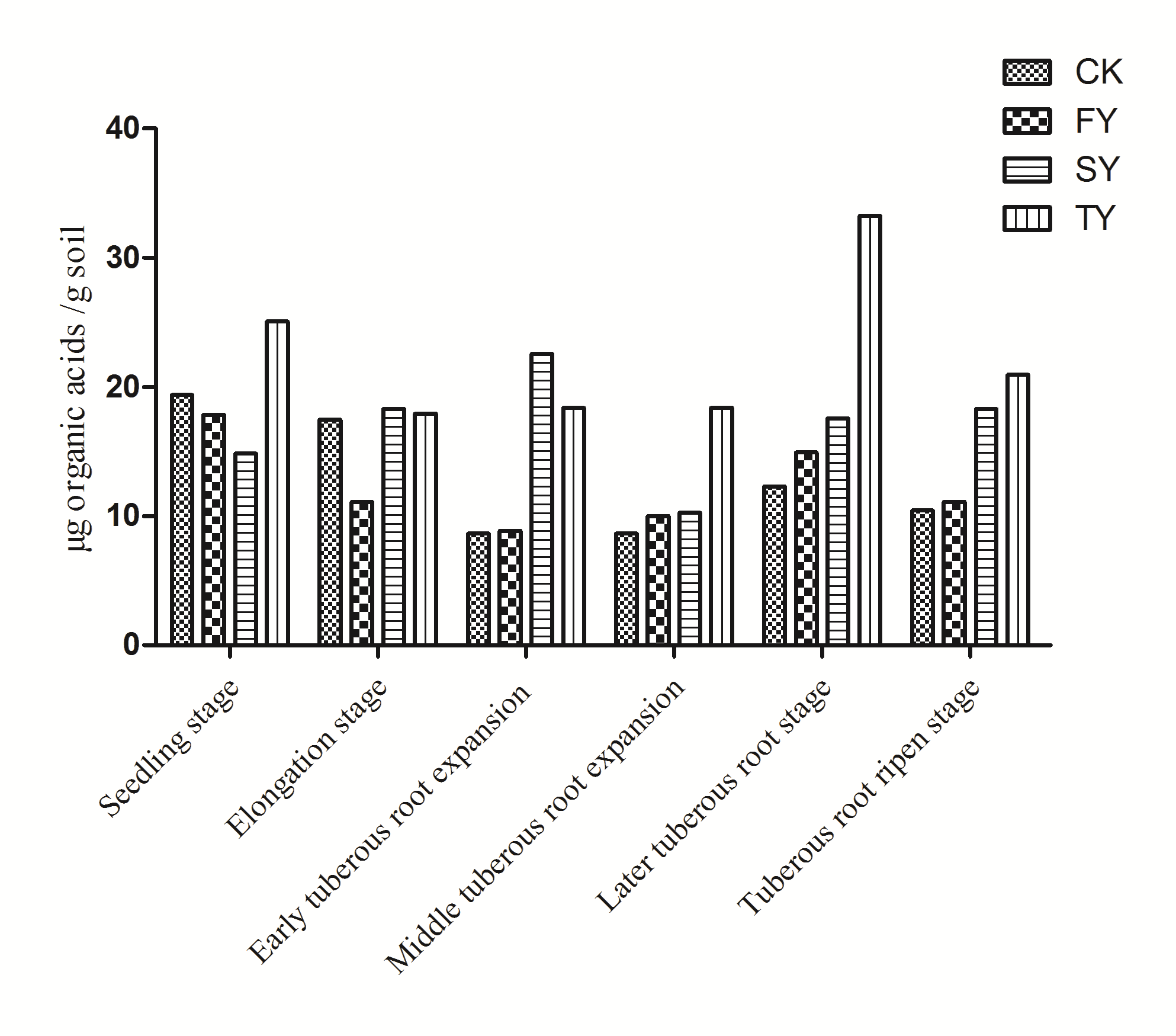


**Figure S2** The total content of organic acid in the rhizosphere soil of *Radix pseudostellariae* sampled at different growth stages in a continuous cropping system. CK represent the control with no *R. pseudostellariae* cultivation. FY, SY, TY represent the newly planted plants, the two-year and three-year monocultured *R. pseudostellariae*, respectively, with plants grown in fields.

**
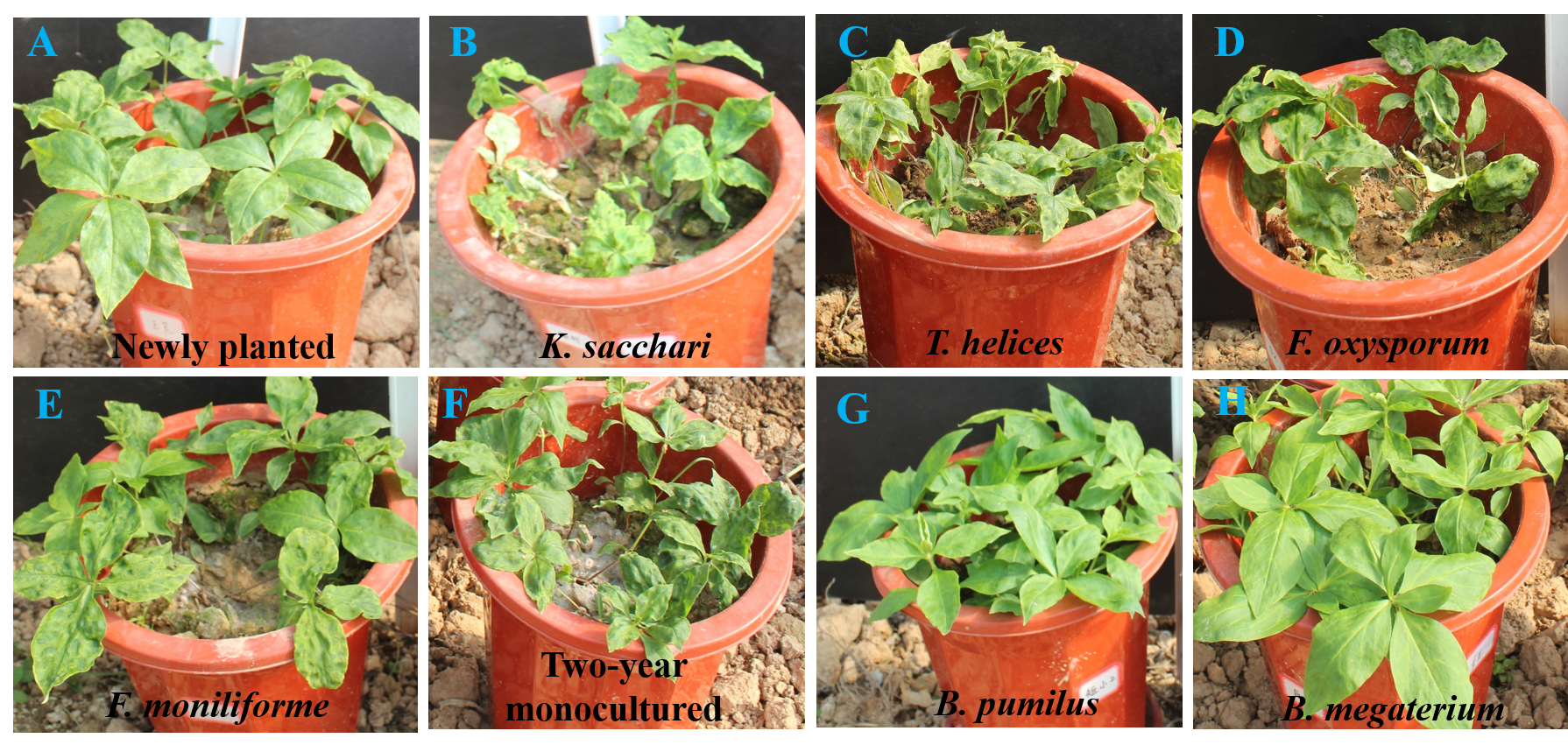
Figure S3** Photographs of *R. pseudostellariae* under different treatments. A, F represent the first cropping and the second cropping year of *R. pseudostellariae*, respectively. B, C, D, E represent the treatment of *Kosakonia sacchari*, *Talaromyces helicus*, *Fusarium oxysporum*, *Fusarium moniliforme* on the first cropping year, respectively. G, H represent the treatment of *Bacillus pumilus*, *Bacillus megaterium* on the second cropping year, respectively.

**
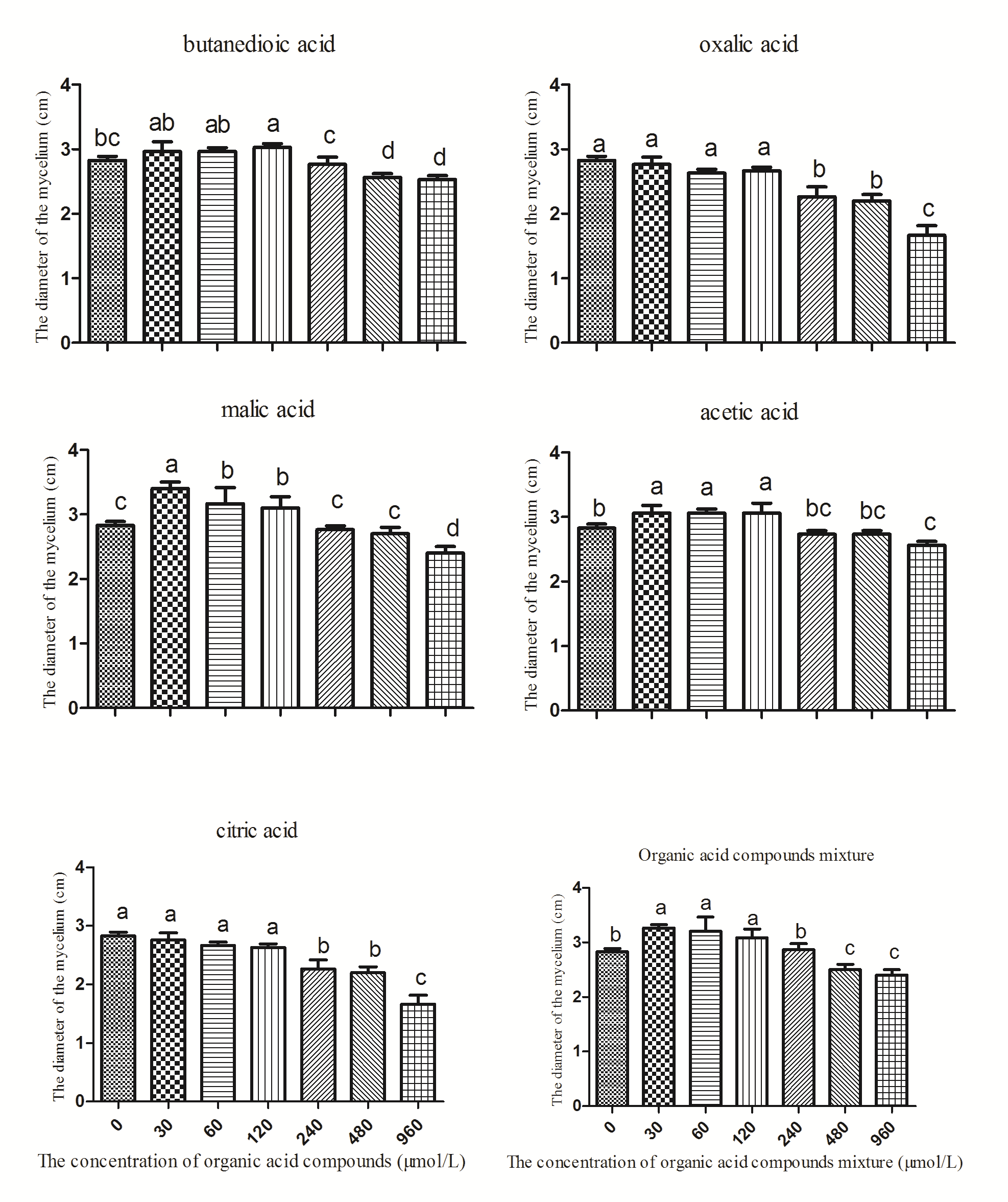
**

**Figure S4** The effects of individual organic acid compounds and their mixture on the mycelial growth of *T. helicus*. Columns with different letters are statistically different (LSD test, p < 0.05).


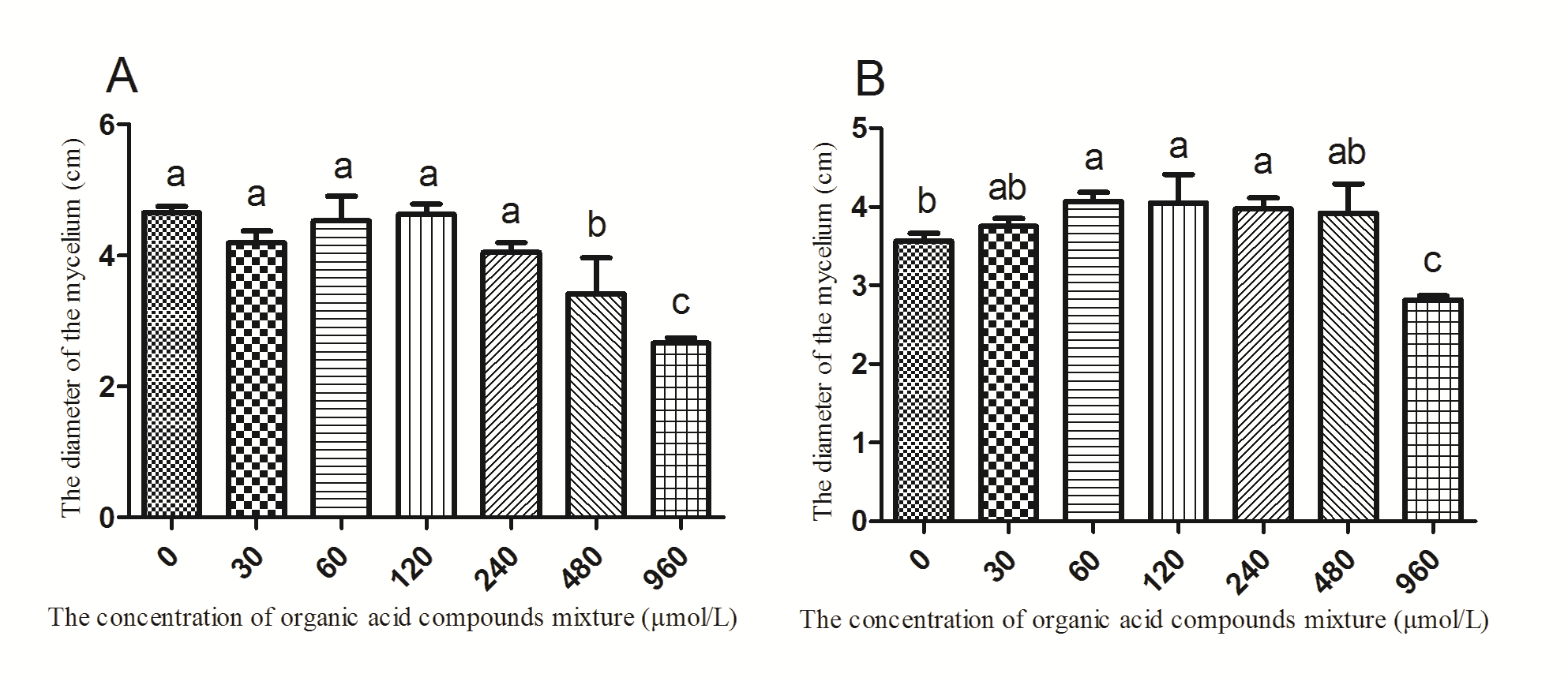


**Figure S5** The effects of an organic acid mixture on the mycelial growth of pathogenic fungi. A represents *F. oxysporum*; B represents *F. moniliforme*. Columns with different letters are statistically different (LSD test, p < 0.05).


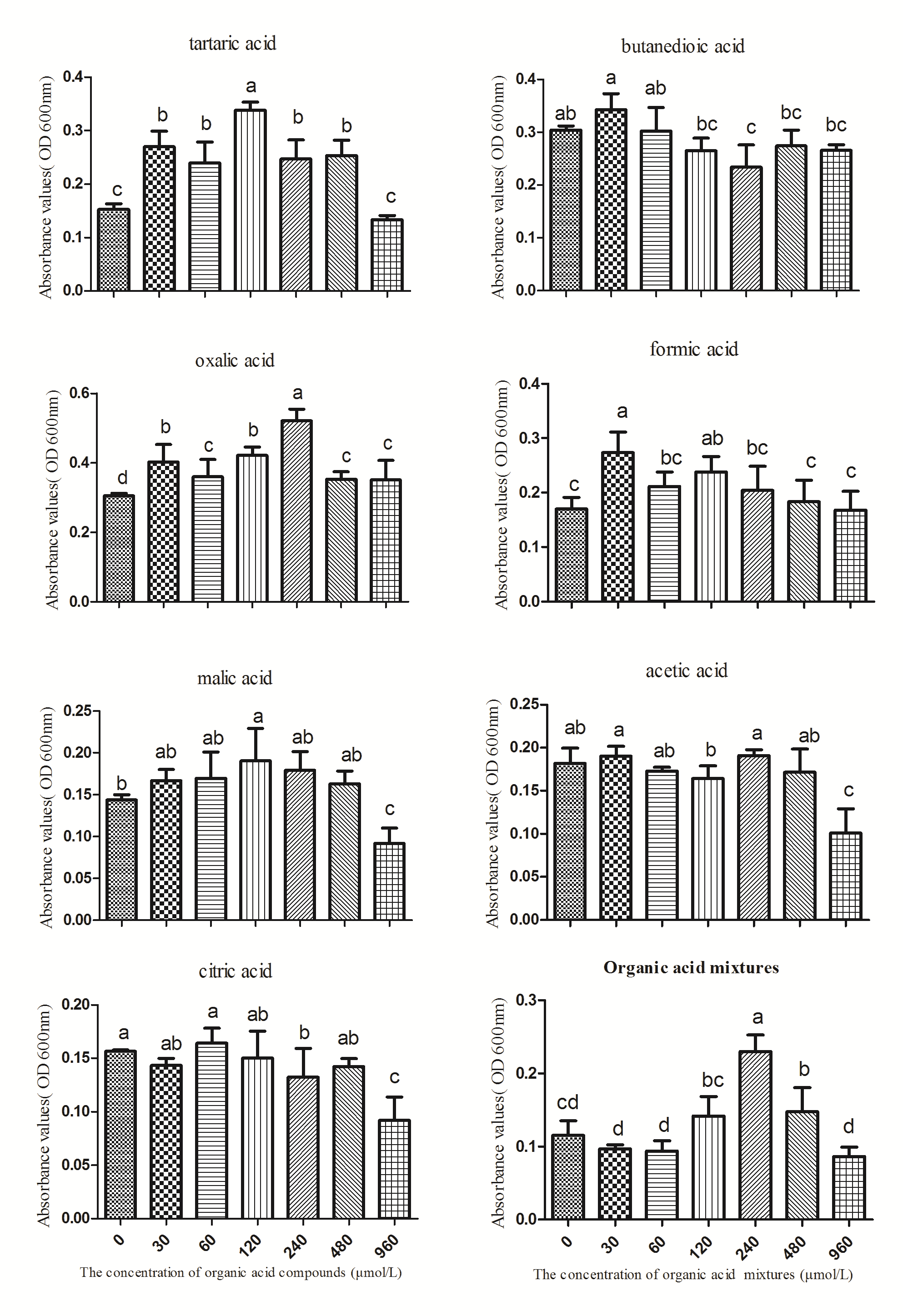


**Figure S6** The effects of single organic acid and the mixture of organic acids on the growth of *K. sacchari*. Columns with different letters are statistically different (LSD test, p < 0.05).


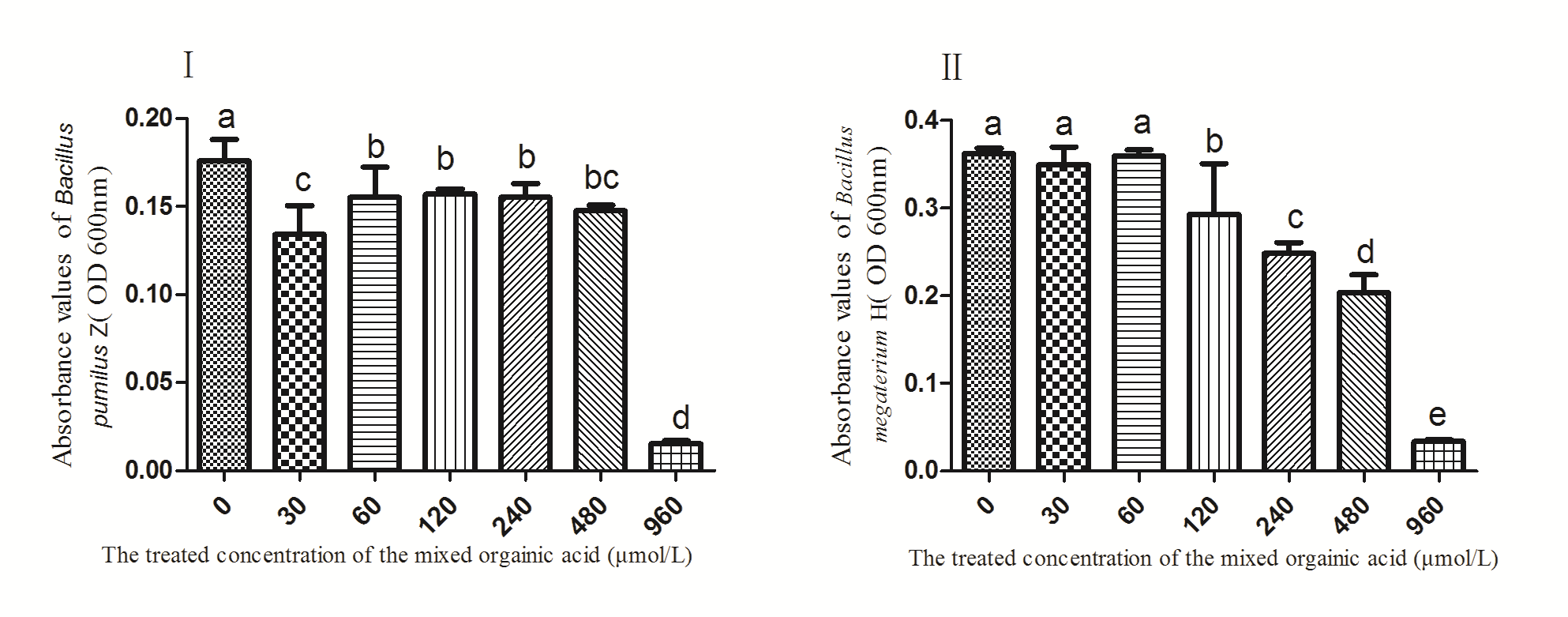


**Figure S7** The effects of the organic acid mixture on the growth of *B. pumilus* and *B. megaterium*. Columns with different letters are statistically different (LSD test, p < 0.05).


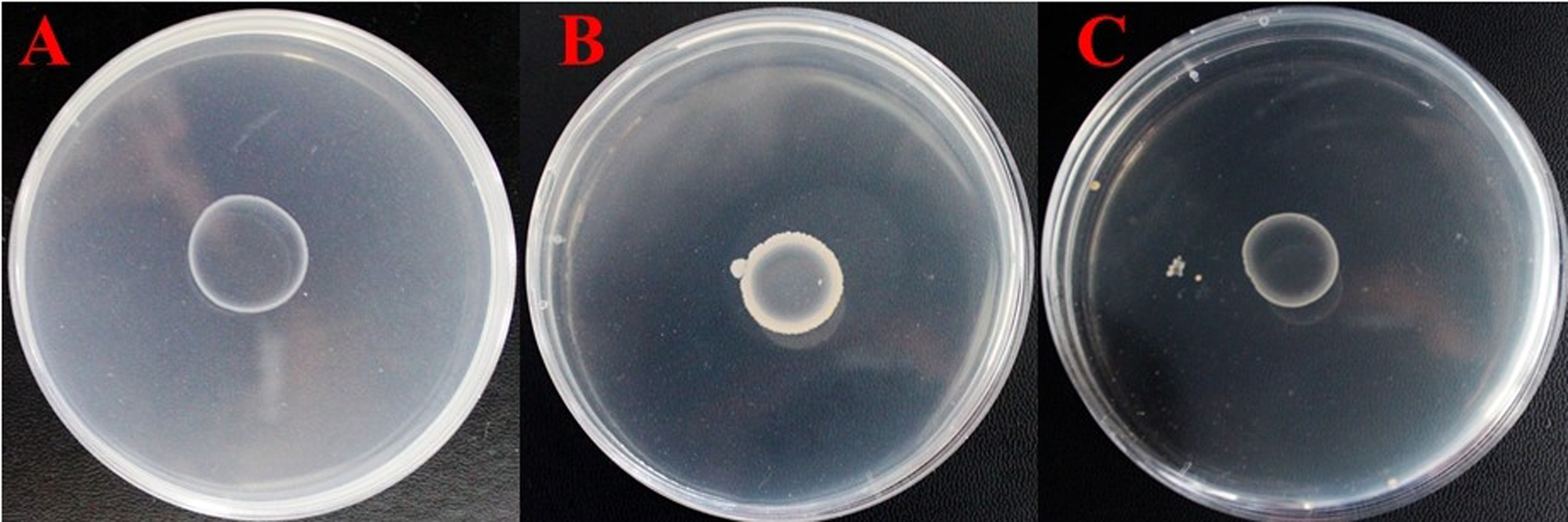


**Figure S8** Chemotactic response of bacteria toward the organic acids. A represents *K. sacchari*; B represents *B. megaterium* and C represents *B. pumilus*.

**
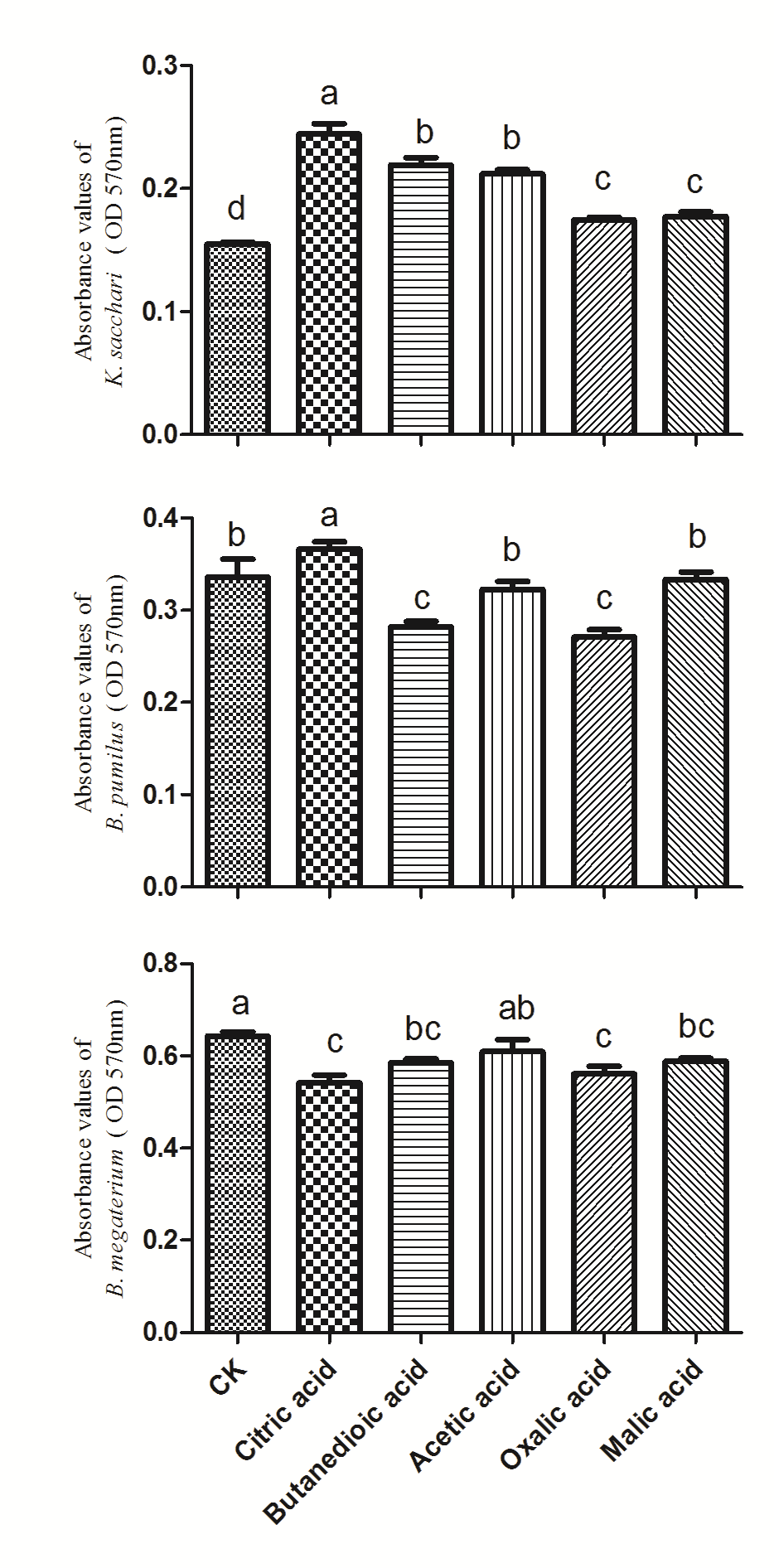
**

**Figure S9** The effects of the different organic acids at 120 μmol/L on biofilm formation of the bacteria. Columns with different letters are statistically different (LSD test, p < 0.05).


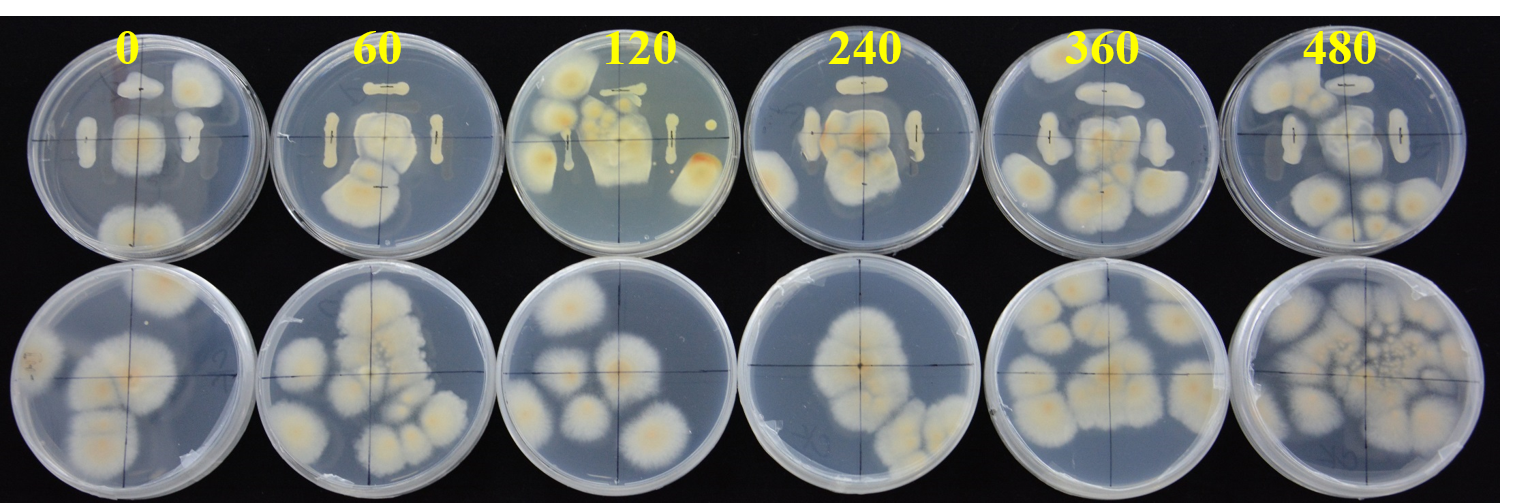


**Figure S10** The dual culture assay between *B. pumilus* and *T. helicus* mediated by organic acids compounds mixture. The numbers represent the concentration of organic acids mixture (μmol/L).

**
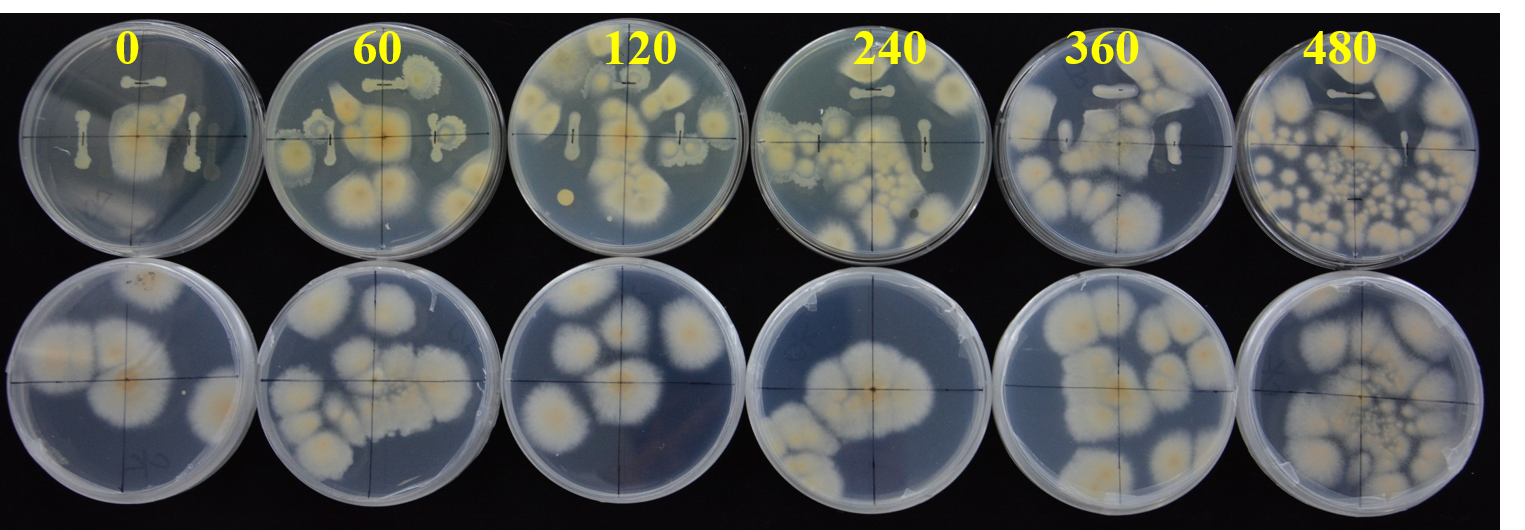
**

**Figure S11** The dual culture assay between *B. megaterium* and *T. helicus* mediated by the mixed organic acids. The numbers represent the concentration of the mixed organic acids (μmol/L).

**
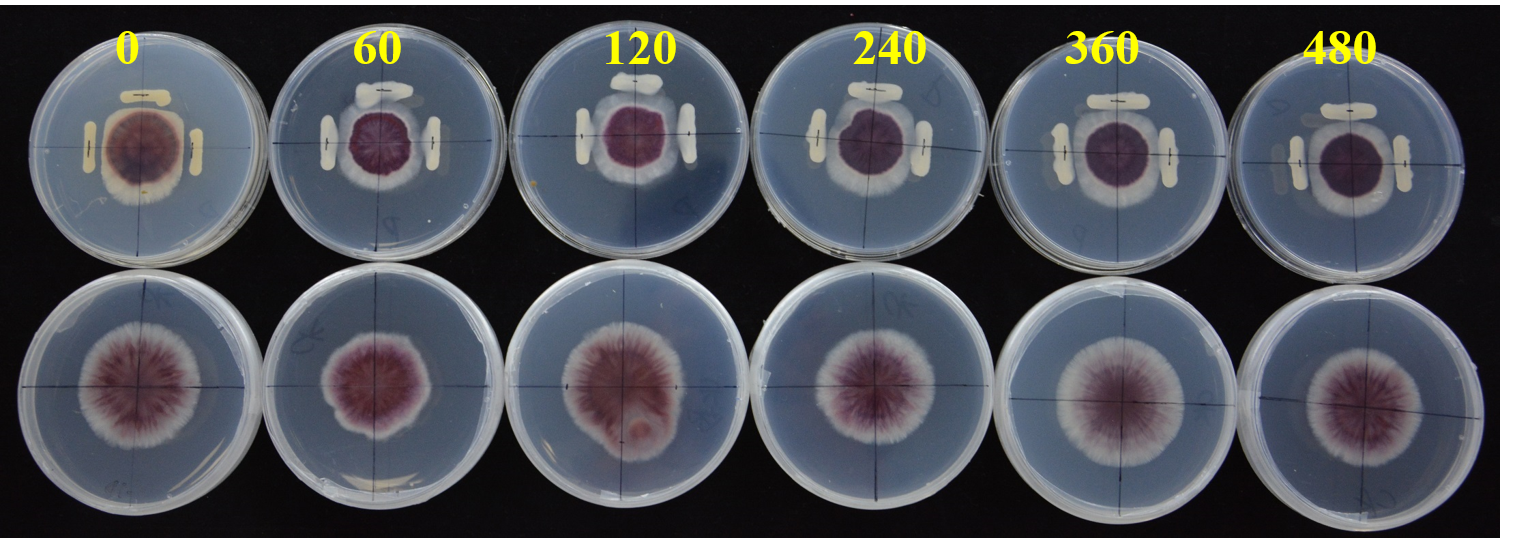
**

**Figure S12** The dual culture assay between *B. pumilus* and *F. oxysporum* mediated by organic acids compounds mixture. The numbers represent the concentration of organic acids mixture (μmol/L).

**
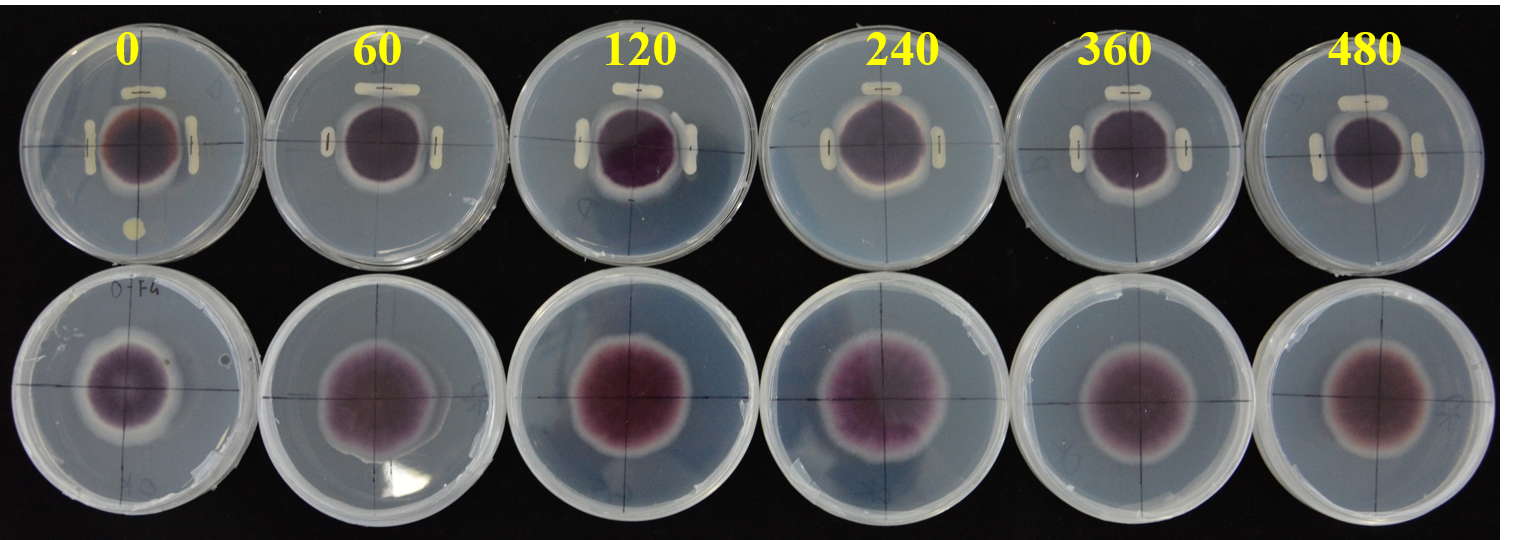
**

**Figure S13** The dual culture assay between *B. pumilus* and *F. moniliforme* mediated by the mixed organic acids. The numbers represent the concentration of the mixed organic acids (μmol/L).
